# Sustained and intensified lacustrine methane cycling during Early Permian climate warming

**DOI:** 10.1038/s41467-022-32438-2

**Published:** 2022-08-18

**Authors:** Funing Sun, Wenxuan Hu, Jian Cao, Xiaolin Wang, Zhirong Zhang, Jahandar Ramezani, Shuzhong Shen

**Affiliations:** 1 State Key Laboratory for Mineral Deposits Research, School of Earth Sciences and Engineering, Nanjing University, Nanjing, 210023 China; 2 Frontiers Science Center for Critical Earth Material Cycling, Nanjing University, Nanjing, 210023 China; 3 Wuxi Institute of Petroleum Geology, Petroleum Exploration and Production Research Institute, SINOPEC, Wuxi, 214126 China; 4 Department of Earth, Atmospheric and Planetary Sciences, Massachusetts Institute of Technology, Cambridge, MA 02139 USA

**Keywords:** Carbon cycle, Palaeoclimate, Stratigraphy

## Abstract

Lakes are a major emitter of the atmospheric greenhouse gas methane (CH_4_); however, their roles in past climate warming episodes remain poorly understood owing to a scarcity of geological records. Here we report the occurrence of sustained and intensified microbial CH_4_ cycling in paleo-Lake Junggar in northwestern China, one of the largest known Phanerozoic lakes, during Early Permian climate warming. High-precision U-Pb geochronology refines the age of the upper Lucaogou Formation to the Artinskian, which marks a major glacial-to-postglacial climate transition. The ^13^C-enriched authigenic dolomites indicate active methanogenesis in the anoxic lake sediments, and ^13^C-depleted hopanes suggest vigorous methanotrophy in the water column. The intensification of CH_4_ cycling coincided with increasing global temperature, as evidenced from elevated continental chemical weathering. Our results suggest that the lacustrine CH_4_ emissions acted as a positive feedback to global warming and contributed to the demise of the Late Paleozoic Ice Age.

## Abstract (web summary)

This study reports the occurrence of sustained and intensified microbial CH_4_ cycling in a giant lake in northwestern China during Early Permian climate warming. Lacustrine CH_4_ emissions may have contributed to the end of the Late Paleozoic Ice Age.

## Introduction

Methane (CH_4_) is a powerful greenhouse gas that has 25-times the global warming potential of carbon dioxide (CO_2_) over a centennial time-scale^1^. Global freshwater CH_4_ emissions, expressed as CO_2_ equivalents, offset at least 25% of the continental greenhouse gas sink^2^. Particularly, lakes are a considerable source of atmospheric CH_4_ and play an important role in the greenhouse gas balance^2,3^. Methane emissions from lakes were estimated as 23–142 Tg CH_4_ yr^–1^ (ref. 4; higher than oceanic emissions), accounting for 4–25% of total global emissions (576 Tg CH_4_ yr^–1^; ref. 5). Understanding the scale and dynamics of CH_4_ emissions from lacustrine ecosystems is therefore fundamental for predicting and reconstructing climate change. Current CH_4_ emissions from lakes have been well documented^2–7^. However, in geological records, lacustrine CH_4_ emissions are poorly understood, and linking lake-source CH_4_ to climate warming in the geological past remains a significant challenge, which substantially hinders our ability to understand the role of this key component of the carbon cycle in ancient climate change and biological systems. Specifically, large uncertainties remain on the metabolic activities of microbial CH_4_ production (methanogens) and consumption (methanotrophs) in large paleo-lakes, which controlled the net CH_4_ emissions to the atmosphere^6–9^.

**Fig. 1:**
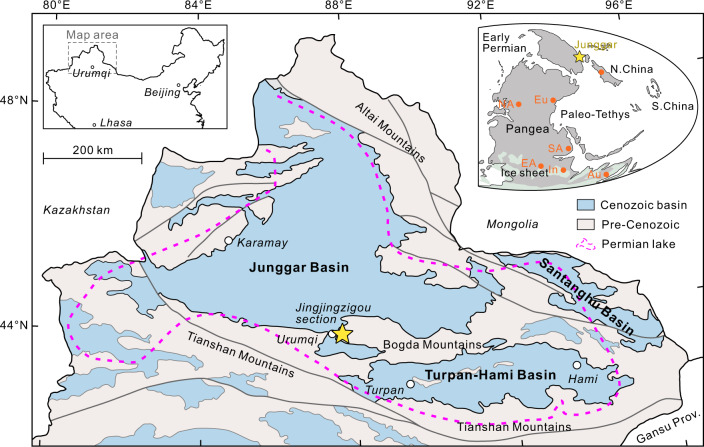
Size and location of paleo-Lake Junggar during the Artinskian (Early Permian). The map shows the locations of the Junggar, Turpan-Hami, and Santanghu basins in northwestern China (modified from ref. 71), with the maximum known extent of Permian lake deposits marked by the purple dashed line^11^. Yellow star represents the location of the Jingjingzigou section. The Early Permian paleogeographic map shows the position of Junggar (adapted after Ronald Blakey, https://deeptimemaps.com, source map © 2016 Colorado Plateau Geosystems Inc). Widespread ice sheet occurred in the Southern Hemisphere. Orange spots represent the locations of other Sakmarian–Artinskian lake systems in the North China block, Southern Arabian plate (SA), Cooper Basin (Australia; Au), Gondwana basins (India; In), East Africa (EA), North America (NA), and Western and Central Europe (Eu). See Supplementary Dataset 8 for associated essential information about these lakes.

The recent discovery of a fossil record of methanogenic archaea in authigenic dolomite from the Permian lacustrine Lucaogou Formation in northwestern China^10^ provides an opportunity to examine geological CH_4_ emissions at the ecosystem level. The extent of the Permian lacustrine deposits in northern Xinjiang covers a total area of ~270,000 km^2^ (900 × 300 km; Fig. 1)^11^, more than three-fold the size of Lake Superior (82,100 km^2^), the largest modern freshwater lake in the world. This Permian lake (referred to as paleo-Lake Junggar in this study) represents one of the largest known Phanerozoic lakes, characterized by the world’s thickest organic-rich lacustrine source rock interval^11–13^.

In this study, high-precision U-Pb zircon geochronology by chemical abrasion–isotope dilution–thermal ionization mass spectrometry (CA-ID-TIMS) and organic carbon isotope (δ^13^C_org_) chemostratigraphy are utilized to constrain the precise depositional age of the Lucaogou Formation. Our results assign this sequence to the Early Permian (Cisuralian), coincident with a critical episode of climate warming in the Artinskian^14^. This time period marks the end of the Late Paleozoic glacial maximum and a climate transition from a long-lived icehouse (340–290 million years ago; Ma)^15^ to a postglacial greenhouse state^16–20^, and it was accompanied by enhanced continental chemical weathering^18,21^, elevated atmospheric partial pressure of CO_2_ (*p*CO_2_)^15,16^, and conspicuous biotic replacement^14^. We further present detailed biogeochemical proxies to reconstruct microbial CH_4_ cycling in paleo-Lake Junggar during this critical climate transition and discuss its possible relevance to the rising global temperatures.

## Results and discussion

### Age and stratigraphic constraints

**Fig. 2:**
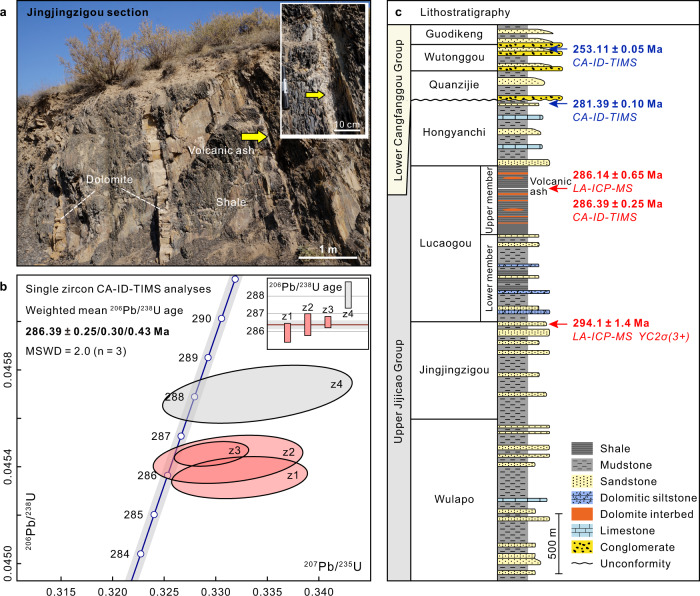
Permian stratigraphy and geochronology of the southern Junggar Basin. **a** Outcrop photograph of organic-rich shale interbedded with dolomite beds and nodules from the upper member of the Lucaogou Formation. The arrow points to the location of the volcanic ash bed (sample VA-1) sampled for zircon geochronology (inset shows a close-up view of the ash bed). **b** Concordia plot and ^206^Pb/^238^U ages of zircons analyzed using the U-Pb CA-ID-TIMS method; excluded analysis z4 shown in gray. Vertical bars represent 2σ analytical uncertainty of individual zircon analyses. **c** Stratigraphic column of the southern Junggar Basin (modified from ref. 11). Arrows indicate stratigraphic positions of dated ash beds (bentonites) and tuffaceous siltstone (blue–published ages of ref. 27; red–new ages presented in this study).

Samples were collected from the Lucaogou Formation at the Jingjingzigou section along the southern margin of the Junggar Basin (Fig. 1 and Supplementary Fig. 1) for zircon U-Pb and geochemical analyses. The Lucaogou Formation can be subdivided into two members. The lower member mainly consists of mudstone, shale, dolomitic siltstone, and dolomite, with minor amounts of gypsum in some layers. The upper member is composed of organic-rich shale interbedded with dolomite beds and nodules without evaporite minerals (Fig. 2a, c; Supplementary Figs. 1 and 2). This sequence reflects the evolution from a relatively shallow evaporative lake to a persistently deep brackish-to-freshwater lacustrine environment (see Supplementary Note 1). Despite decades of sedimentological and geochemical/hydrocarbon research, due to the economic importance of the Lucaogou Formation^11–13,22,23^, the succession lacks any reliable age constraints in the absence of datable volcanic ash beds and biostratigraphically useful fossils^24,25^. Previous detrital zircon U-Pb geochronology obtained by in situ laser ablation–inductively coupled plasma–mass spectrometry (LA-ICP-MS) assigned broad maximum depositional ages of ca. 270–268 Ma^24^ or ca. 261 Ma^26^ to the Lucaogou Formation. However, limited accuracy due to reworked zircons and/or post-crystallization Pb loss can lead to statistically biased results. In addition, a previously published high-precision U-Pb CA-ID-TIMS age of 281.39 ± 0.10 Ma^27^ from the overlying Hongyanchi Formation from the southern Bogda Mountains (Figs. 1 and 2c) resulted in a contradictory stratigraphic framework.

**Fig. 3:**
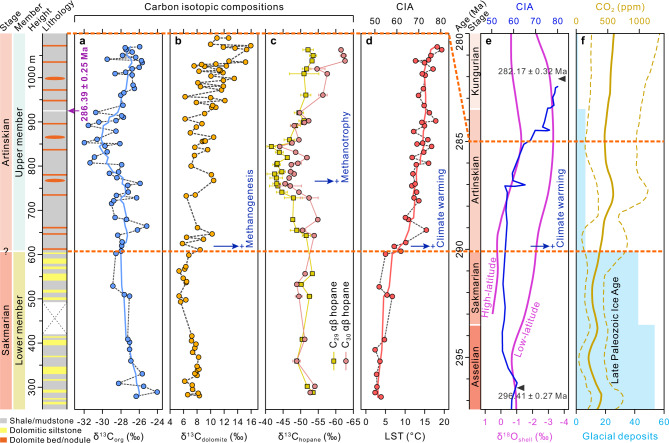
Stable carbon isotopes, estimated land surface temperatures from the Junggar Basin, and comparisons with the Earth system changes during the Early Permian. The different colors in the stratigraphic column indicate changes in the lithology of the Lucaogou Formation. **a** Bulk organic matter δ^13^C_org_ record. **b** Dolomite δ^13^C_carbonate_ record. **c** C_29_ and C_30_ αβ hopane δ^13^C values. Error bars denote one standard deviation between duplicate analyses. **d** Chemical index of alteration (CIA) and land surface temperature (LST) estimates. The curves in (**a**) and (**d**) represent the seven-point moving averages. **e** CIA trend from the glacial to postglacial transition succession in the Karoo Basin of South Africa^21^ with CA-ID-TIMS zircon age constraints^19^ and temporal variations in low-Mg-calcite oxygen isotope (δ^18^O) values from low- and high-latitudinal fossil shells^50,51^. **f** Documented glacial deposits^20^ and reconstructed global atmospheric partial pressure of CO_2_ (*p*CO_2_) curve (75% confidence interval)^15^ during the Early Permian. Timescale from an updated version (2022) of the International Chronostratigraphic Chart^72^.

Here we present high-precision U-Pb zircon age from a volcanic ash bed in the upper, organic-rich member of the Lucaogou Formation (Fig. 2a, b). The 4 cm-thick ash layer interbedded within shales occurs ~925 m above the base of the Lucaogou Formation (Figs. 2c and 3). The sample (VA-1) contains zircons that are small, equant or prismatic, and euhedral, with oscillatory zoning under cathodoluminescence (Supplementary Fig. 3a). The Th/U ratios of the zircon crystals vary from 0.26 to 1.27 (Supplementary Dataset 1). The U-Pb ages determined by LA-ICP-MS have an average 2σ uncertainty of ±5.63 million years (Myr) and are distributed around a well-defined peak, with a weighted mean ^206^Pb/^238^U age of 286.14 ± 0.65 Ma (2σ internal error only; mean-squared weighted deviation [MSWD] = 1.01, *n* = 53; Supplementary Fig. 3a). For further verification, four single zircon grains from this sample were analyzed independently by the CA-ID-TIMS method (average 2σ uncertainty of ±0.55 Myr), with the three youngest analyses constituting a coherent cluster with a weighted mean ^206^Pb/^238^U age of 286.39 ± 0.25/0.30/0.43 Ma (2σ; MSWD = 2.0; Fig. 2b, c and Supplementary Dataset 2). Furthermore, one tuffaceous siltstone (sample TS-1) from the uppermost part of the underlying Jingjingzigou Formation was analyzed using the LA-ICP-MS method. Ninety-four zircon analyses from this sample yielded a wide range of ages, with a weighted mean ^206^Pb/^238^U age of 294.1 ± 1.4 Ma (2σ; MSWD = 1.5; Fig. 2c and Supplementary Fig. 3b) based on 13 youngest analyses (YC2σ[3+]^28^), and this is interpreted as being the maximum constraint on the depositional age. The available radioisotope geochronology collectively places the lower and upper boundaries of the Lucaogou Formation at ca. 294 Ma and ca. 285 Ma, respectively, and constrains its upper shale member to the Artinskian Stage; therefore, the age is significantly older than previous estimates^24–26^.

Organic carbon isotope excursions (CIEs) have been proven to be an effective global stratigraphic correlation proxy^29^. Our results show that a prominent negative δ^13^C_org_ excursion occurs at the upper part of the succession, with a total CIE magnitude of ~3.5‰ (above ~725 m; Fig. 3a and Supplementary Dataset 3). The observed parallel δ^13^C_TLE_ and δ^13^C_Asph_ records from the total lipid extract (TLE) and asphaltene (Asph), after extraction and separation, also exhibit a largely similar negative CIE in shape and magnitude to that of the bulk δ^13^C_org_ record. In addition, all the δ^13^C_*n*-alkane_ records of short-chain *n*-C_19_, mid-chain *n*-C_21_, and long-chain *n*-C_27_ alkanes (Supplementary Dataset 4) display a negative CIE with a magnitude of ~4‰ (Supplementary Fig. 4a). It is considered unlikely that the thermal maturity (early oil window) and proportional changes in the organic matter resulted in the observed CIE in the Lucaogou shales (see Supplementary Note 2; Supplementary Fig. 5). Importantly, under our high-precision CA-ID-TIMS age constraint, the negative CIE is comparable to that recorded in coeval marine brachiopod shells (USA and Russia)^30^, carbonate (South China)^31^, and in coastal strata (North China;^29^ Supplementary Fig. 4). Therefore, the observed parallel CIE signatures in bulk δ^13^C_org_ and δ^13^C_*n*-alkane_ reflect a perturbation of the global carbon cycle during the Artinskian (see Supplementary Note 2).

### Intensified ecosystem-level microbial CH_4_ cycling

Investigating microbial CH_4_ cycling in pre-Holocene environments is challenging owing to the scarcity of diagnostic proxy records; some lipid biomarkers (e.g., glycerol dialkyl glycerol tetraether and archaeol^32,33^) may be invalid with an increase in the thermal maturity of the organic matter. In this study, we present the distinctive δ^13^C records of authigenic dolomites and hopanes (bacterial-derived biomarkers) from the Lucaogou Formation, which provide new insights into the metabolic activities of methanogens and methanotrophs in the lake ecosystem during the Early Permian.

Dolomite beds and nodules in the upper member (Fig. 2a) have very positive δ^13^C values (+5.8 to +16.0‰) that are significantly higher than the δ^13^C values (+5.3 to +8.3‰) of the dolomite in the lower member (Fig. 3b and Supplementary Dataset 5). Several mechanisms have been proposed for ^13^C enrichment in inorganic C pools^34–36^. Of these, the Rayleigh distillation of volatile CO_2_ under highly evaporative conditions^34^ would not have been effective in paleo-Lake Junggar, as the upper member was not deposited in a hypersaline environment^12,13^. In addition, the photosynthetic fixation of CO_2_ during productivity blooms cannot explain the positive values, because this process usually only enriches δ^13^C values by +2 to +3‰^35^, and this Permian lake was not eutrophic^13^. Such positive δ^13^C signatures have been recently attributed to authigenic dolomite precipitation associated with microbial methanogenesis^10^, and it is likely that some dolomite samples with less positive δ^13^C and lower δ^18^O values in the upper member have been influenced by subsequent diagenesis^36^ (Supplementary Fig. 6). Microbial methanogenesis is geochemically characterized by significant C isotopic fractionation, generating ^13^C-depleted biogenic CH_4_ (δ^13^C as low as −60 to −110‰)^37^ and ^13^C-enriched CO_2_ (δ^13^C up to +15‰ or higher)^36^. Such isotopically heavy CO_2_ acted as a substantial C source and was incorporated into the authigenic dolomite. The closest modern analogues of these dolomites, commonly observed in organic carbon-rich continental margin sediments, have been documented in the Gulf of California^38^ and along the Peru Margin^39^, where methanogenesis is highly active in oceanic sediments. Thus, the ^13^C-enriched authigenic dolomites presented here are a fingerprint of biogenic CH_4_ production in lake sediments.

Putative methanogen microfossils have been found in these ^13^C-enriched dolomites from the adjacent Hongyanchi section, and their abundances show a positive correlation with δ^13^C values^10^. The elevated δ^13^C signature of the dolomites can therefore be used to trace changes in methanogenesis. In the current study, the dolomite δ^13^C values show an overall increasing trend from the bottom to the top within the succession (Fig. 3b). The dolomite beds and nodules that are marked by high δ^13^C values occur above ~610 m. Most importantly, the more abundant strongly ^13^C-enriched dolomites occur within the upper part of the Lucaogou Formation (Fig. 3b), indicating a higher methanogenic rate and/or an expanded methanogen community in the anoxic lake sediments during this period. In contrast, the absence of exceptionally ^13^C-depleted authigenic dolomite in the studied section (δ^13^C_carbonate_ values typically <−30‰; C largely derived from biogenic CH_4_)^40^ suggests that the anaerobic oxidation of methane was not an important process occurring in the anoxic sediments at that time. Therefore, large amounts of CH_4_ produced in bottom sediments escaped substantial consumption and were emitted into the overlying water column; as such, they would have provided substrates for aerobic methanotrophs.

An earlier comprehensive study^13^ revealed that the saturated hydrocarbon fraction from Lucaogou shales was depleted in ^13^C, and this possibly indicates the presence of hopanes derived from methanotrophic bacteria. In this study, we conducted a compound-specific C isotope analysis of hopanes. Hopanoids are not exclusive to methanotrophs, but their stable C isotopic compositions can be used to assess specific methanotroph contributions^33,41–45^. Methanotrophic bacteria use biogenic CH_4_ as a carbon source for the biosynthesis of membrane lipids (e.g., hopanoids) that are highly ^13^C-depleted. Our results show that the hopanoids in Lucaogou shales are dominated by C_30_ 17α,21β-hopane and C_29_ 17α,21β-norhopane (Supplementary Fig. 7 and Supplementary Note 1). The hopane δ^13^C values remain low in all samples analyzed, ranging from −44.1 to −62.6‰ for C_30_ 17α,21β-hopane and −41.6 to −53.6‰ for C_29_ 17α,21β-hopane (Fig. 3c). The δ^13^C values in these two compounds yield a positive correlation (Supplementary Fig. 5d), indicating that they have a similar bacterial community source. Their corresponding 17β,21α(H) isomers are also characterized by similar low isotopic signatures (Supplementary Dataset 4). The δ^13^C values of the hopanes are markedly lower than those observed in the co-occurring bulk organic matter (−24.0 to −32.0‰; Fig. 3a, c) and *n*-alkanes (*n*-C_21_: −33.0 to −38.1‰). Such ^13^C-depleted hopanoids also appear in some modern/Holocene (e.g., Lake Rotsee, Switzerland^33^) and Eocene (e.g., Green River Formation, USA^46^) lake systems, where aerobic CH_4_ oxidation by methanotrophic bacteria was prevalent in the water column. Here we conducted a survey of hopanoid δ^13^C values from 19 lakes (283 data points; δ^13^C_hopanoid_ ranging from −22.2 to −71.9‰; Supplementary Fig. 8 and Supplementary Dataset 6). The data compilation (see Supplementary Note 3 for data overview) suggests that hopanoid δ^13^C values below −40‰ are indicative of a pronounced aerobic methanotroph contribution to these compounds (>10–20%; calculated from a C isotopic mass-balance approach;^33^ see Methods).

Hopanoid δ^13^C values can be used to trace the temporal changes in aerobic CH_4_ oxidation^33,42,44,45^. In this study, the consistently low hopane δ^13^C values (<−40‰) throughout the section indicate that methanotrophic activity within the lake was sustained and vigorous, particularly during the late depositional stage of the Lucaogou Formation (Fig. 3c). Specifically, in the lower member, the suitably low hopane δ^13^C values (ca. −50‰) and the less positive dolomite C isotopic signatures (<+8.5‰; Fig. 3b) indicate mild-to-moderately active methanotrophy and methanogenesis. In the upper member, however, the coupling between highly ^13^C-depleted hopanes and ^13^C-enriched authigenic dolomites suggests that both methanotrophs and methanogens thrived in the lake biosphere. In particular, above ~750 m, there is a persistent and obvious decrease in hopane δ^13^C values of >10‰ (from ca. −46 to −63‰ for C_30_ hopane and from ca. −43 to −54‰ for C_29_ hopane; Fig. 3c). The lowest values within the uppermost stratigraphic interval are among the most ^13^C-depleted reported in the C_30_ and C_29_ hopanes for lacustrine systems (Supplementary Fig. 8). These isotopic signatures indicate that substantially intensified CH_4_ oxidation occurred in the water column, which closely coincided with elevated CH_4_ production in the sediments, as indicated by a temporal increase in dolomite δ^13^C values (Fig. 3b, c). The combined evidence from both authigenic dolomite and molecular fossil (hopane) suggests that an intensification of the microbial CH_4_ cycling occurred during the Artinskian age. Furthermore, active CH_4_ cycling had a wide geographical distribution in paleo-Lake Junggar, with evidence of similar ^13^C-depleted hopanes also documented in the Lucaogou shales from the adjacent Sangonghe section^22^ and the Santanghu Basin^23^, hundreds of kilometers from the studied area (Fig. 1). Based upon our estimated depositional duration (ca. 9 Myr) of the Lucaogou Formation, the intensified microbial CH_4_ cycling persisted for at least ca. 3–5 Myr. To our knowledge, such a long-term dynamic of lacustrine CH_4_ cycling in the Earth’s history has not been previously and directly revealed.

### Positive feedback to Artinskian climate warming

To investigate the relationship between temperature and microbial CH_4_ cycling, we used the chemical index of alteration (CIA)^47^ to reconstruct changes in the land surface temperature (LST;^18,48^ see Methods). The collected samples were not affected by K-metasomatism, and their uniform Ti/Al ratios indicate no changes in provenance^48^ (Supplementary Fig. 9 and Supplementary Dataset 7), and they thus provide a reliable record of climate variation. The CIA profiles show an increase from 50–55 in the lower member to 65–75 in the upper member, suggesting a rapid rise in the estimated LSTs from ~4 °C (Sakmarian) to ~14 °C (Artinskian; Fig. 3d). Overall, the pronounced progression toward higher CIA values, combined with the alternative chemical index of weathering (CIW;^49^ Supplementary Fig. 9), indicates a shift toward warmer conditions^14,18,48^. This record is consistent (within age uncertainties) with an independently derived CIA trend in a contemporaneous succession from the Karoo Basin of South Africa^19,21^ (Fig. 3e). A cross-basin correlation revealed that a significant increase in CIA (temperature) globally began near the Sakmarian–Artinskian boundary (ca. 290 Ma)^18^. This major climate transition can be further corroborated by a coincident decrease in δ^18^O values from both low- and high-latitudinal fossil shells composed of low-Mg calcite^50,51^ (Fig. 3e). Therefore, the elevated continental weathering in this study reflects a global climate warming signal (i.e., the Artinskian Warming Event^14^), which developed contemporaneously with the intensification of CH_4_ cycling in paleo-Lake Junggar (Fig. 3).

Higher temperatures may have stimulated methanogenesis in lake sediments, supporting a temperature control on CH_4_ cycling at the ecosystem level^8,42,52^. It has been proposed that the metabolic responses of methanogens are particularly sensitive to increases in temperature^8,52^. Since the predominant microbial methanogenesis occurred in the shallow sediment columns^53^, it would be expected that the increase in atmospheric temperature warmed the sediments and subsequently facilitated methanogenic activity. Additionally, under global warming, enhanced continental weathering (Fig. 3d) may have increased riverine nutrient influx and aquatic productivity in lakes, thereby resulting in increased substrate (e.g., acetate and H_2_/CO_2_; ref. 6) availability for methanogenesis^8^. However, methanotrophy is known to have a more positive effect on substrate (i.e., CH_4_) availability than temperature^8^, and the intensified CH_4_ consumption observed in the top part of the Lucaogou Formation (mid-Artinskian) was almost certainly a response to an increased CH_4_ substrate supply for methanotrophs (Fig. 3).

The balance between methanogenesis and methanotrophy ultimately controlled the amount of CH_4_ released into the atmosphere^6,8,9^. Nonetheless, if a warming-induced increase in CH_4_ production exceeds the increase in CH_4_ oxidation, an increase in net CH_4_ emissions is expected, and this provides potential positive feedback to climate warming. Indeed, owing to the different temperature sensitivities of methanogens and methanotrophs^8,9^, warming would increase CH_4_ emissions, which has been extensively observed in both modern freshwater ecosystems^7,52,54^ and laboratory incubations^8,9,52^. For example, experimental warming of artificial ponds has suggested a disproportionate increase in methanogenesis over methanotrophy^9^. Although aerobic methanotrophs did oxidize more CH_4_, but not enough to offset the greater warming-induced CH_4_ production^9^. Methane fluxes from lake ecosystems exhibit a temperature dependence^8,52,54^. The prevailing paradigm of the exponential response of CH_4_ emissions to temperature^7,8,52,54^ can be extrapolated to ancient lake systems, and the total CH_4_ emissions from paleo-Lake Junggar could potentially have increased by several-fold in response to Artinskian climate warming. Applying the average CH_4_ flux (total 31.6 Tg CH_4_ yr^−^^1^ in areas spanning 1,330,264 km^2^; i.e., 65 mg CH_4_ m^–2^ d^–1^)^2^ from modern lakes at similar latitudes to paleo-Lake Junggar (paleolatitude of 39–43°N)^13^, the flux was roughly estimated as 6.4 Tg CH_4_ yr^–1^ (accounting for 5–28% of annual lake CH_4_ emissions in the modern world^4^), and a total amount of ~19,200 Gt CH_4_ was emitted from this Early Permian lake (~270,000 km^2^; ref. 11; herein conservatively calculated using 3 Myr).

Although there is only evidence for intensified CH_4_ cycling in paleo-Lake Junggar (Fig. 1), this still provides a useful analogue for similar environments having responses to Artinskian (Early Permian) climate warming. In this respect, several contemporaneous lake systems (see Fig. 1 and Supplementary Dataset 8 for the locations of these lakes and associated essential information) may also be CH_4_ emission hotspots. However, accurate assessments of global CH_4_ emissions require clear constraints relating to the contemporaneous lake area, distribution, and environmental factors, and these are beyond the scope of this study. Nonetheless, large-scale lacustrine CH_4_ emissions would have acted as a positive feedback to Artinskian global warming and a critical mechanism for deriving carbon cycle perturbations. During this time period (after 290 Ma)^15^, the demise of the Late Paleozoic Ice Age (LPIA) was supported by a 6-fold drop in documented glacial deposits (Fig. 3f)^20^ and the full deglaciation in south-central Gondwana by 282 Ma^19^, representing one of the most prominent and enigmatic climate transitions in the Earth’s Phanerozoic history. Previous studies have demonstrated that widespread deglaciation was synchronous with an increase in atmospheric *p*CO_2_ (Fig. 3f)^15^ derived from volcanic eruptions (e.g., Tarim, Panjal, and Zaduo large igneous provinces)^15,29^, and this provides an evidence regarding the strong link between CO_2_ and glaciation. In addition to the contribution of CO_2_ (refs. 15–17) and potential methane clathrate release^55^, our results suggest that the injection of the terrestrial greenhouse gas CH_4_ into the atmosphere may have facilitated the demise of the LPIA and played a direct role in forcing the turnover from a long-lived icehouse to a greenhouse world.

In summary, this study investigated currently unexplored lacustrine ecosystem-level microbial CH_4_ cycling records, including methanogenesis and methanotrophy, in pre-Cenozoic sedimentary archives. Our results suggest that sustained and intensified CH_4_ cycling, as a response to Artinskian (Early Permian) climate warming, occurred in paleo-Lake Junggar. The release of the greenhouse gas CH_4_ from large paleo-lakes to the atmosphere could have provided a direct positive feedback to ancient global warming, at least during the Early Permian, which should improve our understanding of its role in near-future climate change within a warming-but-glaciated world.

## Methods

### Samples

The samples analyzed in this study were collected from the Jingjingzigou section (43°47′30″ N, 87°45′12″ E) in the Urumqi area, Xinjiang, Northwest China (Fig. 1). The outcrop mainly occurs along road cuts, which are generally continuous (Supplementary Fig. 1). One volcanic ash sample from the upper Lucaogou Formation and one tuffaceous siltstone sample from the uppermost part of the underlying Jingjingzigou Formation were collected for zircon U-Pb dating. Additionally, 67 shale and 82 dolomitic rocks were collected for petrographic and geochemical analyses. The samples were visually examined to ensure that they were fresh.

### Microscopic analysis

Polished thin-sections were examined using a Nikon Eclipse LV100N POL fluorescence microscope. Freshly broken chips coated with platinum were observed using a Carl Zeiss Supra 55 field-emission scanning electron microscope (FE-SEM), equipped with an energy dispersive X-Ray spectrometer, at the State Key Laboratory for Mineral Deposits Research, Nanjing University (MiDeR-NJU), China. Analyses were carried out at an acceleration voltage of 5 kV and a beam current of 10 nA.

### Zircon U-Pb geochronology

Zircon grains were separated using standard density and magnetic separation techniques, mounted in epoxy, and polished to a half section. Cathodoluminescence (CL) images were obtained using a JEOL JSM-7000F SEM. Zircon U-Pb dating was performed on an Agilent 7900 ICP-MS, equipped with a Resolution SE 193 nm laser ablation (LA) system at Nanjing Hongchuang Exploration Technology Service Co., Ltd. The detailed tuning parameters were similar to those described in ref. 56. Analyses were conducted with a spot diameter of 30 μm and a repetition rate of 5 Hz. U-Pb fractionation was corrected using zircon standard 91,500, and the accuracy was controlled using zircon standard GEMOC GJ-1. Fifteen analyses of the GJ-1 zircon in this study yielded a weighted mean ^206^Pb/^238^U age of 602.2 ± 1.8 Ma (2σ; MSWD = 1.5), which is in good agreement (within a 2σ error) with the reference TIMS age (599.8 ± 1.7 Ma; 2σ)^57^. Exported data were reduced offline using the Iolite software package^58^ and diagrams were created with the ISOPLOT/Ex program (ver. 4.15)^59^. The ^206^Pb/^238^U dates for the zircon grains were selected for all grains <1000 Ma^28^. The maximum depositional age was estimated from the weighted mean age of the youngest cluster of more than three statistically overlapping analyses (YC2σ[3+])^28^.

U-Pb analyses were conducted on single zircons using CA-ID-TIMS method at the Massachusetts Institute of Technology Isotope Laboratory, following the general procedures described in ref. 60. Prior to dissolution and analysis, the selected zircon crystals were thermally annealed at 900 °C for 60 h and subsequently leached in 29 M HF inside high-pressure vessels at 210 °C for 12 h to minimize the effects of radiation-induced Pb loss in the crystals^61^. The chemically abraded grains were thoroughly rinsed and fluxed to remove the leachates before being spiked with the EARTHTIME ET535 mixed ^205^Pb-^233^U-^235^U trace solution^62,63^ and dissolved completely in 29 M HF at 210 °C for 48 h. Both U and Pb were isolated using an HCl-based anion exchange column chemistry procedure, deposited onto outgassed rhenium filaments with a silica gel emitter solution, and analyzed on an IsotopX X62 multi-collector thermal ionization mass spectrometer equipped with a Daly photomultiplier ion-counting system. Data reduction, age calculation and error propagation were carried out using the Tripoli and ET_Redux software^64,65^. The sample age was derived from the weighted mean ^206^Pb/^238^U date obtained from a coherent cluster of the youngest zircon after excluding older (detrital or xenocrystic) outliers and is reported at 95% confidence level. Uncertainty is reported as ± X/Y/Z Ma, where X is the internal uncertainty (2σ) in the absence of all external errors, Y incorporates X and the tracer calibration error, and Z includes Y as well as the decay constant errors of ref. 66.

### Total organic C, total N, and Rock-Eval pyrolysis analyses

The powdered shale samples were treated with 2 M HCl for 24 h to remove carbonate, rinsed with distilled water to neutral pH, and then oven-dried at 60 °C for ~72 h. The total organic carbon (TOC) and total nitrogen (TN) contents were measured by an Elementar Vario MACRO CHNS elemental analyzer. Rock-Eval pyrolysis analysis was performed with ~100 mg powdered samples that were heated gradually in an inert atmosphere. The pyrolysis parameters included free hydrocarbon (S_1_), hydrocarbons cracked from kerogen (S_2_), CO_2_ released from organic matter (S_3_), and the temperature of the maximum pyrolyzate yield (T_max_).

### Bulk organic C isotope

Approximately 0.2–2 mg (based on TOC values) of de-carbonated powdered sample was weighed into tin capsules for bulk δ^13^C_org_ analyses. The δ^13^C_org_ values were measured by a Thermo Scientific Flash 2000 Elemental Analyzer coupled to a Thermo Scientific MAT 253 isotope ratio mass spectrometer (IRMS) via a Conflo IV open split interface at MiDeR-NJU. The organic C isotopic compositions of TLE and Asph (after extraction and separation procedures) were also measured and were expressed as δ^13^C_TLE_ and δ^13^C_Asph_, respectively. The reproducibility and accuracy of organic C isotopes were evaluated by measuring standards (USGS 40) between sample measurements. The isotopic ratios were reported as δ^13^C values relative to the V-PDB standard with a precision of ±0.2‰ or better.

### Biomarker and compound-specific C isotope analyses

Approximately 50 g of powdered shale sample was Soxhlet extracted for 72 h using dichloromethane (DCM):methanol (93:7; v/v). The obtained TLE was concentrated using rotary evaporation and then de-asphalted with *n*-hexane, followed by filtration. The Asph-free fractions were then separated into three fractions using chromatographic columns pre-filled with alumina:silica gel (1:3; w/w; activated at 120 °C for 12 h) via elution with *n*-hexane (saturated fraction), DCM:*n*-hexane (2:1; v/v; aromatic fraction), and ethanol (polar fraction). The retrieved saturated fraction of each sample was further treated with a ZSM-5 molecular sieve to isolate the branched and cyclic alkanes, following the method described in ref. 43. The molecular sieve was activated at 120 °C for 2 h and added to a Pasteur pipette plugged with a small piece of pre-extracted cotton wool. Approximately 2 mg of the saturated fraction was dissolved in ~0.5 mL of cyclohexane and transferred to the ZSM-5-filled Pasteur column. Subsequently, the branched/cyclic hydrocarbon fraction was eluted with 3 column bed volumes of cyclohexane (~6 mL) and concentrated under a gentle nitrogen flow.

Both the original saturated and ZMS-5 treated branched/cyclic fractions were analyzed by gas chromatography–mass spectrometry (GC-MS) and gas chromatography–isotope ratio mass spectrometry (GC-IRMS) at the State Key Laboratory of Shale Oil and Gas Enrichment Mechanisms and Effective Development of SINOPEC, China. GC-MS analysis was conducted using an Agilent 7890B-5977A GC-MS equipped with a 60 m × 0.25 mm i.d. J&W DB-5MS capillary column (film thickness of 0.25 µm). Samples were injected into the injector held at 290 °C in splitless mode; helium was used as the carrier gas. The GC oven was initially held at 80 °C for 3 min, increased to 230 °C at 3 °C min^–1^, and finally programmed to 310 °C at 2 °C min^–1^, followed by a 20 min isothermal hold. The MS was operated at an ion source energy of 70 eV with full-scan mode.

The GC-IRMS analysis was performed on an Agilent 7890 GC interfaced to a Thermo Scientific Delta V Plus IRMS. The GC was fitted with a DB-5 capillary column (30 m × 0.25 mm i.d., film thickness of 0.25 μm). The initial temperature of the GC oven was 80 °C held for 5 min; it was then programmed to 320 °C at 2 °C min^–1^ with an isothermal hold of 15 min. Sample injection was conducted in splitless mode at 305 °C. The isotopic ratios were reported as δ^13^C values relative to the V-PDB standard. Samples were measured in duplicate with a reproducibility of typically <1–2‰. The instrument stability was monitored via a regular analysis of an in-house gas (CO_2_) standard with a known δ^13^C value, and the long-term precision was found to be better than ±0.5‰.

### Carbonate C and O isotope analysis

The bulk-rock C and O isotopes of dolomite were measured using a Thermo Finnigan Delta V Plus continuous flow IRMS at MiDeR-NJU. Approximately 80–120 µg of each powdered sample was reacted with orthophosphoric acid at 70 °C for >2 h in a continuous flow sample preparation device (Gas Bench II) that was connected to the IRMS. The isotopic ratios were reported as δ^13^C and δ^18^O values relative to the V-PDB reference. The internal precision (1 SD) was less than ±0.1‰ and the external precision was better than ±0.5‰.

### Shale elemental analysis

The carbonate-free powdered shale samples were prepared for whole-rock major elemental concentration analysis using a Thermo Scientific ARL 9900 X-Ray fluorescence spectrometer (XRF) at MiDeR-NJU. A mixture of 1 g of powdered sample and 11 g of co-solvent (Li_2_B_4_O_7_/LiBO_2_/LiBr: 49.75%/49.75%/0.50%) was melted to a glass disk in a platinum crucible at 1050 °C. The analyses were performed on tablets with an accelerating voltage of 40 kV, a beam current of 70 mA, and a count time of 20 s for each element. The analytical precision was better than ±1% for elemental concentrations of >1% and ±10% for elemental concentrations of <1%.

### Estimating contribution of methanotrophic bacteria to hopanoids

To estimate the contribution of methanotrophs to hopanoids, we applied a simple C isotopic mass-balance approach described in ref. 33,

1 $${f}_{{{{{{{\rm{CH}}}}}}}_{4}-{{{{{\rm{C}}}}}}}=\frac{{\updelta}^{13}{{{{{{\rm{C}}}}}}}_{{{{{{\rm{hopanoid}}}}}}-{{{{{\rm{sample}}}}}}}-{{{\updelta }}}^{13}{{{{{{\rm{C}}}}}}}_{{{{{{\rm{non}}}}}}-{{{{{{\rm{CH}}}}}}}_{4}-{{{{{\rm{C}}}}}}}}{{{\updelta }}^{13}{{{{{{\rm{C}}}}}}}_{{{{{{{\rm{CH}}}}}}}_{4}-{{{{{\rm{C}}}}}}}-{{{\updelta }}}^{13}{{{{{{\rm{C}}}}}}}_{{{{{{\rm{non}}}}}}-{{{{{{\rm{CH}}}}}}}_{4}-{{{{{\rm{C}}}}}}}}\times 100,$$

where ${f}_{{{{{{{\rm{CH}}}}}}}_{4}-{{{{{\rm{C}}}}}}}$ is the fraction of the CH_4_-carbon sources in the hopanoids (%; i.e., the fraction of the hopanoids originating from methanotrophic bacteria), ${{{\updelta }}}^{13}{{{{{{\rm{C}}}}}}}_{{{{{{\rm{hopanoid}}}}}}-{{{{{\rm{sample}}}}}}}$ is the measured δ^13^C values of the hopanoids in a given sample, ${{{\updelta }}}^{13}{{{{{{\rm{C}}}}}}}_{{{{{{\rm{non}}}}}}-{{{{{{\rm{CH}}}}}}}_{4}-{{{{{\rm{C}}}}}}}$ is the C isotopic composition of the non-CH_4_ carbon end member (i.e., the organic C of hopanoids that is 0% CH_4_ derived), ${{{\updelta }}}^{13}{{{{{{\rm{C}}}}}}}_{{{{{{{\rm{CH}}}}}}}_{4}-{{{{{\rm{C}}}}}}}$ is the C isotopic composition of the CH_4_ carbon end member (i.e., the organic C of hopanoids that is 100% CH_4_ derived). For end member values, a ${{{\updelta }}}^{13}{{{{{{\rm{C}}}}}}}_{{{{{{\rm{non}}}}}}-{{{{{{\rm{CH}}}}}}}_{4}-{{{{{\rm{C}}}}}}}$ value of –30‰ and a ${{{\updelta }}}^{13}{{{{{{\rm{C}}}}}}}_{{{{{{{\rm{CH}}}}}}}_{4}-{{{{{\rm{C}}}}}}}$ value of −73 to −109‰ were used^33^. According to Eq. (1), the proposed baseline of hopanoid δ^13^C values of −40‰ indicated that approximately 10–20% of these compounds were derived from methanotrophic bacteria. In addition, hopanoid δ^13^C values of −60‰ showed that approximately 40–70% of the hopanoids originated from methanotrophs (Supplementary Fig. 8).

### Paleoweathering indices and paleotemperature estimation

The CIA^47^ has been widely used to evaluate the degree of chemical weathering. The CIA value was calculated using molecular proportions as follows,

2 $${{{{{\rm{CIA}}}}}}=[{{{{{{\rm{Al}}}}}}}_{2}{{{{{{\rm{O}}}}}}}_{3}/({{{{{{\rm{Al}}}}}}}_{2}{{{{{{\rm{O}}}}}}}_{3}+{{{{{{\rm{CaO}}}}}}}^{\ast }+{{{{{{\rm{Na}}}}}}}_{2}{{{{{\rm{O}}}}}}+{{{{{{\rm{K}}}}}}}_{2}{{{{{\rm{O}}}}}})]\times 100,$$

where CaO* only represents the CaO in the silicate fractions^47^. The correction methods for CaO* followed those described in refs. 67, 68. High CIA values (i.e., those approaching 100) indicate a greater weathering intensity caused by the removal of readily soluble cations (e.g., Ca^2+^, Na^+^, and K^+^) relative to the stable residual cations (e.g., Al^3+^ and Ti^4+^) during chemical weathering^69^. In contrast, low CIA values reflect less chemical alteration; fresh crystalline rocks are characterized by CIA values of ca. 40–50 (ref. 68). In addition, K-metasomatism can influence the K_2_O contents, resulting in lower CIA values. The effects of K-metasomatism were assessed using A-CN-K (Al_2_O_3_-CaO* + Na_2_O-K_2_O) diagrams^69^.

To avoid the effects of K-metasomatism, an alternative chemical index of weathering (i.e., CIW^49^) was developed that eliminates K_2_O from the CIA equation. The CIW proxy has been tested in numerous studies^48,68^ and is expressed as follows,

3 $${{{{{\rm{CIW}}}}}}=[{{{{{{\rm{Al}}}}}}}_{2}{{{{{{\rm{O}}}}}}}_{3}/({{{{{{\rm{Al}}}}}}}_{2}{{{{{{\rm{O}}}}}}}_{3}+{{{{{{\rm{CaO}}}}}}}^{\ast }+{{{{{{\rm{Na}}}}}}}_{2}{{{{{\rm{O}}}}}})]\times 100,$$

where CaO* also represents silicate-bound CaO^49,67^. To assess the possible impacts that changes in the sediment provenance have on the chemical weathering indices, Ti/Al ratios were applied (wt.% presented in this study)^48^.

The chemical weathering of silicate rocks is closely related to climatic conditions. The CIA values of modern suspended particulate matters from large global rivers are particularly more sensitive to LSTs, the latitude of the river mouth, and the soil depth in the drainage basin than other factors^70^. Furthermore, a relationship between the CIA and LST as a paleothermometer was quantified as follows^18^,

4 $${{{{{\rm{T}}}}}} \, ({\!\,}^\circ{{{{{\rm{C}}}}}})=0.56\times {{{{{\rm{CIA}}}}}} - 25.7( {R}^{2}=0.50),$$

which is robust over a temperature range of 3–25 °C and has an uncertainty of approximately ±5 °C, corresponding to a CIA range of approximately 50–90. This LST estimation has been applied to quantify paleotemperatures of several Permian and Triassic terrestrial successions^18,48^. In this study, we used the CIA and LST proxies to assess changes in chemical weathering and temperature.

### Reporting summary

Further information on research design is available in the Nature Research Reporting Summary linked to this article.

## Supplementary information

Supplementary Information

Description of Additional Supplementary Files

Supplementary Data 1

Supplementary Data 2

Supplementary Data 3

Supplementary Data 4

Supplementary Data 5

Supplementary Data 6

Supplementary Data 7

Supplementary Data 8

Reporting Summary

## Data Availability

The geochronological and geochemical data generated in this study are provided in the Supplementary Dataset files.
