# Supplementary material for: Sustained and intensified lacustrine methane cycling during Early Permian climate warming
Source: Nat Commun. 2022 Aug 18;13:4856. doi: 10.1038/s41467-022-32438-2 (PMC9388690; doi:10.1038/s41467-022-32438-2)
Supplement: Supplementary file 1 — Supplementary Information [file 41467_2022_32438_MOESM1_ESM.pdf]

Supplementary Information for

Sustained and intensified lacustrine methane cycling during Early Permian climate warming

Funing Sun[1,2], Wenxuan Hu[1,2]*, Jian Cao[1,2], Xiaolin Wang[1,2], Zhirong Zhang[3], Jahandar Ramezani[4], Shuzhong Shen[1,2]

[1]State Key Laboratory for Mineral Deposits Research, School of Earth Sciences and Engineering, Nanjing University, Nanjing 210023, China

[2]Frontiers Science Center for Critical Earth Material Cycling, Nanjing University, Nanjing 210023, China

[3]Wuxi Institute of Petroleum Geology, Petroleum Exploration and Production Research Institute, SINOPEC, Wuxi 214126, China

[4]Department of Earth, Atmospheric and Planetary Sciences, Massachusetts Institute of Technology, Cambridge, MA 02139, USA

*Corresponding author at: School of Earth Sciences and Engineering, Nanjing University, 163 Xianlin Avenue, Nanjing 210023, China. E-mail: huwx@nju.edu.cn

This PDF file includes:

Supplementary Notes 1–3

Supplementary Figures 1–9

Supplementary References

Supplementary Note 1. Results and Interpretation

Sedimentology and petrography

The Lucaogou Formation is a mixed clastic–dolomitic sequence predominantly composed of organic-rich shales/mudstones interbedded with fine-grained dolomitic rocks[1,2] (Fig. 2a,c; Supplementary Figs. 1 and 2). This unit was deposited in a stratified and anoxic moderately saline-to-brackish lake environment[3,4]. This formation contains fossils of freshwater fish, such as *Tienshaniscus longipterus*, *Chichia gracilis*, and *Turfania taoshuyuanensis*, and diverse non-marine ostracods and molluscs[3]. The faunal assemblages include *Anthraconautia*, *Kelameilia*, and *Tomiella*, and the palynological assemblage is *Cordaitina uralensis–Vittatina scottii–Hamiapollenites mutabilis*[5].

The lower member of the Lucaogou Formation mainly consists of nonlaminated mudstone and dolomitic siltstone to siltstone (Figs. 2c and 3; Supplementary Fig. 1), with little amounts of less-laminated shale and micritic dolomite. Some gypsum and desiccation cracks can be observed, which provide evidence for evaporative conditions. The siltstone, dolomitic siltstone, and silty dolomite commonly range from centimeters to sub-meters in thickness (Supplementary Fig. 1e,f), usually exhibiting plane laminate and low-angle cross-stratification. The dolomite minerals in the dolomitic rocks are typically fine-crystalline, distributed between the surrounding clay and silt (Supplementary Fig. 2c,d). The sedimentary features suggest marginal to semi-deep lake environments.

The upper member is composed of well-laminated shale interbedded with micrite dolomite beds and nodules (Figs. 2a,c and 3; Supplementary Fig. 1). The shale is typically black and laminated at a millimeter scale, forming alternating organic matter layers and siliceous-rich layers (Supplementary Fig. 2a). Fluorescence imaging showed that lamalginites and terrestrial land-plant debris are common in the shale (Supplementary Fig. 2b). Dolomites commonly occur as thin interlayers or nodules (a few centimeters to decimeters thick)

interbedded within the black organic-rich shales (Fig. 2a; Supplementary Fig. 1g–i). They are microcrystalline (typically < 10 μm) and contain dolomite microspheres (Supplementary Fig. 2e,f), which were interpreted as methanogen microfossils[2]. Evidence for evaporite minerals and subaerial exposure is absent. Thus, it is concluded that the upper member is the basinal facies deposited in a semi-deep to deep lake environment.

Zircon U-Pb geochronology

In the field, the volcanic ash (sample VA-1) is yellowish-white and occurs as a thin interlayer (thickness of ~4-cm; Fig. 2a; see Supplementary Fig. 1a for the accurate location) within the organic-rich shales in the upper member of the Lucaogou Formation. Most of the zircon crystals separated from this sample have a euhedral equant or prismatic morphology, vary from 50 to 100 μm in size, and are characterized by oscillatory zoning under cathodoluminescence (CL). Their Th/U ratios range from 0.26 to 1.27, with an average value of 0.58 (Supplementary Dataset 1). From this sample, 53 concordant laser ablation–inductively coupled plasma–mass spectrometry (LA-ICP-MS) U-Pb age analyses display a single peak distribution, yielding a weighted mean $^{206}Pb/^{238}U$ age of 286.14 ± 0.65 Ma (million years ago; 2σ; mean-squared weighted deviation [MSWD] = 1.01; Supplementary Fig. 3a). Furthermore, four zircon grains from this sample were analyzed using the chemical abrasion–isotope dilution–thermal ionization mass spectrometry (CA-ID-TIMS) method, and the data are presented in Supplementary Dataset 2. Three out of the four zircons constituted a coherent cluster with a weighted mean $^{206}Pb/^{238}U$ age of 286.39 ± 0.25/0.30/0.43 Ma (2σ; MSWD = 2.0; Fig. 2b). A slightly older zircon grain (z4) with an age of 288.06 ± 0.74 Ma, interpreted as detrital, was excluded from date calculation.

The tuffaceous siltstone (sample TS-1; Supplementary Fig. 1d) is gray and massive. This sample yielded a heterogeneous population of colorless to light pink and rounded to

prismatic zircons, commonly with lengths < 100 μm. CL imaging revealed that most grains have concentric oscillatory zoning while some display less notable zoning with uniform brightness. The Th/U ratios range from 0.30 to 1.98 (average value of 0.73; Supplementary Dataset 1). The LA-ICP-MS analysis yielded 94 concordant U-Pb dates with a wide range of ages. The $^{206}Pb/^{238}U$ ages range from 504 to 286 Ma, with a dominant (91%) population between 348 to 286 Ma. The youngest 13 analyses define a coherent cluster with a weighted mean $^{206}Pb/^{238}U$ age of 294.1 ± 1.4 Ma (2σ; MSWD = 1.5), which is interpreted as the maximum depositional age (YC2σ[3+]; mean age of the youngest three or more grains that overlap in age at 2σ; Supplementary Fig. 3b).

Organic C isotope vs. hydrogen index, C/N, total organic carbon

The bulk organic carbon isotope ($\delta^{13}C_{org}$) values of the Lucaogou shales range from –24.0 to –32.0‰, with an average value of –27.9‰ (Supplementary Dataset 3). There is a negative carbon isotope excursion (CIE) above ~725 m, with an amplitude of ~3.5‰ (Fig. 3a). At the top of the succession, the isotopic values return to higher values of approximately –27‰, comparable to those in the pre-CIE interval. The $\delta^{13}C_{TLE}$ and $\delta^{13}C_{Asph}$ values of the total lipid extract (TLE) and asphaltene (Asph) vary from –28.9 to –33.4‰ and –27.8 to –32.4‰, respectively, slightly lower than the corresponding $\delta^{13}C_{org}$ records. They also exhibit a largely similar negative CIE to the $\delta^{13}C_{org}$ records throughout the succession (Supplementary Fig. 4a). All of the samples are organic-rich (total organic carbon [TOC] values of 1.3–19.8 wt.%, average value of 6.6 wt.%). The C/N ratios vary from 2.0 to 19.1 (average value of 9.4) and the hydrogen index (HI) values range from 100 to 660 mg HC/g TOC, with an average value of 346 mg HC/g TOC (Supplementary Dataset 3). The temperatures of the maximum pyrolyzate yield (T_{max}) vary from 429 °C to 447 °C, with an average value of 439 °C, indicating that they are within the early oil widow. No significant

correlations exist between the $\delta^{13}C_{org}$ values and HI (Supplementary Fig. 5a) and C/N ratio values (Supplementary Fig. 5b). There is only a weak correlation between the $\delta^{13}C_{org}$ and TOC values (Supplementary Fig. 5c).

Dolomite C and O isotopes

The dolomite $\delta^{13}C$ values in the lower member are characterized by little variations, from +5.3 to +8.3‰ (Supplementary Fig. 6), and are slightly positive than those recorded in the Early Permian marine carbonates from the Yangtze Platform, South China (ca. 0 to +6‰)[6]. In contrast, the dolomite $\delta^{13}C$ values in the upper member show relatively large variations and significantly positive values, ranging from +5.8 to +16.0‰ (Supplementary Dataset 5 and Supplementary Fig. 6). An overall increasing trend in the $\delta^{13}C$ values does occur from the bottom to the top of the stratigraphic section (Fig. 3b). There is no significant difference in the $\delta^{18}O$ values between the dolomites from the lower and upper members of the Lucaogou Formation (Supplementary Fig. 6). As the dolomite is mostly microcrystalline, late diagenesis likely had a relatively minor influence on isotopic compositions. However, some dolomite samples, characterized by less positive $\delta^{13}C$ and lower $\delta^{18}O$ values and an observed positive correlation in the upper member, possibly formed via authigenic processes with a progressive burial depth during early diagenesis.

Molecular compositions and compound-specific C isotopes

The samples commonly contain a suite of *n*-alkanes from C_{17} to C_{33} without an odd-even C number preference. The C_{19}–C_{23} *n*-alkanes are the predominate compounds, with a maximum at C_{21} in most samples (Supplementary Fig. 7a). The major isoprenoids are pristane (Pr) and phytane (Ph), with more abundant Ph (Pr/Ph ratio typically ranging between

0.4 and 0.9). The samples also include small amounts of steranes. Both C_{30} 17α,21β-hopane and C_{29} 17α,21β-norhopane appear to be the dominant hopanes, together with minor amounts of 17β,21α(H) isomers. The C_{31}–C_{34} homohopane abundances decrease with an increasing carbon number. Tm (17α-22,29,30-trisnorhopane) and Ts (18α-22,29,30-trisnorneohopane) are also present, with Ts/(Ts + Tm) ratios ranging from 0.24 to 0.45. The sterane epimerization ratios, i.e., C_{29} 20S/(20S + 20R) and C_{29} αββ/(αββ+ααα), range from 0.41 to 0.52 and 0.22 to 0.45, respectively (Supplementary Dataset 4). These biomarker maturity ratios indicate an early oil window thermal maturity for the organic matter and there is no systemic change in thermal maturity throughout the section.

To constrain the effect that molecular sieving has on isotopic fractionation, we compared the $\delta^{13}C$ values of the hopanes before and after ZSM-5 treatment using a representative sample at 898.61 m, characterized by a high abundance of hopanes and trace amounts of steranes. The results showed that the procedure appeared to not impact the isotopic composition (Supplementary Fig. 7). Some hopanoids (i.e., C_{29} and C_{30} 17α,21β-hopanes and their corresponding 17β,21α(H) isomers) had good resolution in the gas chromatography–isotope ratio mass spectrometry (GC-IRMS) chromatogram, such that they yielded reliable isotopic signatures. Supplementary Dataset 4 lists the measured $\delta^{13}C$ values of the *n*-alkanes (before ZSM-5 treatment) and hopanes (after ZSM-5 treatment). The $\delta^{13}C$ values of short-chain *n*-C_{19}, mid-chain *n*-C_{21}, and long-chain *n*-C_{27} vary from –31.9 to –36.9‰, –33.0 to –38.1‰, and –30.2 to –38.6‰, respectively. There is a negative $\delta^{13}C_{n\text{-alkane}}$ excursion at the upper part of the succession above 725 m, with an amplitude of ~4‰ (Supplementary Fig. 4a). In contrast, the hopanes have significantly lower $\delta^{13}C$ values than the *n*-alkanes. C_{30} 17α,21β-hopane, the most abundant hopane, yields an average $\delta^{13}C$ value of –51.4‰ (ranging from –44.1 to –62.6‰), similar to the $\delta^{13}C$ values of C_{29} 17α,21β-hopane, with an average value of –48.3‰ (ranging from –41.6 to –53.6‰). The $\delta^{13}C$ values

of the C_{30} and C_{29} 17α,21β-hopanes are positively correlated (R^2 = 0.528; Supplementary Fig. 5d). Below ~750 m, the $\delta^{13}C$ values of the C_{30} and C_{29} 17α,21β-hopanes have minor variations, with an average value of –51.5‰ and –50.1‰, respectively. At the upper part of the succession (above ~750 m), the values decrease progressively to approximately –63‰ and –54‰ for the C_{30} and C_{29} 17α,21β-hopanes, respectively (Fig. 3c). The 17β,21α(H) isomers, i.e., C_{30} and C_{29} 17β,21α-hopanes also have relatively ^{13}C-depleted values, ranging from –45.3 to –62.5‰ and –41.2 to –55.7‰, respectively (Supplementary Dataset 4); their isotopic profiles exhibit similar temporal trends throughout the succession.

Paleoweathering index and land surface temperature

As shown in the molar A-CN-K ternary diagram[7], the samples correspond to the expected weathering trend based on the regional protolith source, i.e., Late Paleozoic volcanic rocks from the Junggar Basin, with an average chemical index of alteration (CIA) value of 48 (ref. [8]; Supplementary Fig. 9), suggesting that the CIA values were not affected by K-metasomatism. The CIA and chemical index of weathering (CIW) values range from 50 to 81 and 54 to 92, respectively (Supplementary Dataset 7), with a strong covariation (R^2 = 0.98; Supplementary Fig. 9b). The CIA values of the lower member of the Lucaogou Formation average ca. 50–55, whereas samples in the upper member mainly range from 65 to 75 (Fig. 3d). The CIW values tend to be slightly higher than the CIA values, with a similar temporal trend to CIA. The Ti/Al ratios are uniform throughout the stratigraphic section, with an average value of 0.05 ± 0.003 (1 SD; Supplementary Fig. 9c), indicating that the shifts in the CIA and CIW cannot be attributed to changes in sediment provenance. Thus, the profiles of the weathering indices (CIA and CIW) provide reliable records of the variation in paleoweathering in the studied area.

According to the CIA-derived temperature estimates[9], the land surface temperatures (LSTs) show relatively large variations, ranging from 2.4 °C to 19.4 °C. A sharp rise in the LST of ~10 °C (from ~4 °C to ~14 °C) occurred in the transition from the lower to upper members (Fig. 3d). However, even if the absolute calculated temperatures are not strictly constrained, we suggest that the shift in climate warming recorded by our enhanced chemical weathering indices (CIA and CIW) is reliable and can be compared with the CIA trends in many contemporaneous successions (as shown in Fig. 2 in ref. [9]), such as that from the Karoo Basin in South Africa[10]. In addition, the decrease in the $\delta^{18}O$ values, as recorded by both low- and high-latitudinal fossil shells composed of low-Mg calcite, suggest an increase in the seawater temperature during the Artinskian[11,12]. Thus, the consistent terrestrial and marine temperature estimates indicate that climate warming was prevalent at that time (i.e., the Artinskian Warming Event[13]).

Supplementary Note 2. $\delta^{13}C_{org}$ Stratigraphy and Correlation

A prominent negative $\delta^{13}C_{org}$ excursion occurs at the upper part of the studied succession (Supplementary Fig. 4a). However, several factors in a given basin can affect the organic C isotopic compositions. The T_{max} (429–447 °C; Supplementary Dataset 3) and biomarker maturity parameters (Supplementary Dataset 4) indicate an early oil window thermal maturity of the organic matter, which is unlikely to have induced such large changes in the $\delta^{13}C_{org}$ signatures[14,15]. In addition, it has been proposed that the two sources from terrestrial versus lacustrine organic matter show distinctive $\delta^{13}C_{org}$, HI, and C/N signatures[15,16]; crucially, however, there is no apparent relationship between $\delta^{13}C_{org}$ and the HI and C/N ratios in the Lucaogou shales, despite a poor correlation with TOC (Supplementary Fig. 5a–c). Therefore, it is unlikely that proportional changes in the organic matter from terrestrial versus lacustrine environments also resulted in the observed CIE. For

further verification, we report compound-specific C isotope analyses of *n*-alkanes with different chain lengths (Supplementary Dataset 4). These compounds were derived, in a large part, from the cracking of parent molecules in kerogen. The results show that all the $\delta^{13}C_{n\text{-alkane}}$ records of short-chain *n*-C_{19}, mid-chain *n*-C_{21}, and long-chain *n*-C_{27} alkanes display a negative CIE with a magnitude of ~4‰, which is similar to the bulk $\delta^{13}C_{org}$ record (Supplementary Fig. 4a). Under our high-precision CA-ID-TIMS age constraint, this negative CIE is consistent with that recorded in coeval marine brachiopod shells from the U.S. Midcontinent and the Russian Platform[17], carbonates from the Yangtze Platform in South China[6], and in coastal strata from North China Platform[18] (Supplementary Fig. 4a−c). Thus, the observed parallel CIE signatures in bulk $\delta^{13}C_{org}$ and $\delta^{13}C_{n\text{-alkane}}$ probably reflect a global carbon cycle perturbation during the Artinskian. The negative CIEs in both $\delta^{13}C_{org}$ (this study and another[18]) and $\delta^{13}C_{carbonate}$[6,17] coincided with a short-term peak in atmospheric partial pressure of carbon dioxide (pCO_2; ~600 ppm) during the mid-Artinskian (ca. 289–286 Ma; Supplementary Fig. 4)[19], which indicates that the carbon cycle perturbation may be linked to the elevated atmospheric CO_2 level during this time interval. In addition, a negative valley with the $\delta^{13}C_{carbonate}$ values below −10‰ within the Artinskian Stage was observed in the Dal'ny Tulkas section in southern Urals, Russia (Supplementary Fig. 4b), and this may have been superimposed on the regional signal linked to isotopic refractionation by microbial chemosynthetic processes[20].

Supplementary Note 3. Compilation of Hopanoid $\delta^{13}C$ Values in Lake Systems

The stable C isotopic composition of hopanoids has been used to study modern and past lake biogeochemistry involved in methane (CH_4) cycling[21,22] and the source of organic matter[23–25]. These compounds include hopane, norhopane, hop-17(21)-ene, hop-22(29)-ene (diploptene), neohop-13(18)-ene, moret-22(29)-ene (moretene), and homohopanoic acid.

Here we undertook a survey of hopanoid $\delta^{13}C$ values (n = 283 data points) from 19 lake systems spanning the modern-Holocene, Miocene, Oligocene, and Eocene (Supplementary Dataset 6). Based upon this survey, we explored the utility of hopanoid $\delta^{13}C$ signatures as indicators for methanotroph input and CH_4 cycling in natural archives.

Our global dataset shows that hopanoids exhibit large variations in their $\delta^{13}C$ values, ranging from –22.2 to –71.9‰, with an average value of –49.0‰ (Supplementary Fig. 8a and Supplementary Dataset 6). The C_{29} and C_{30} hopanes, some of the most abundant in ancient lacustrine sequences, yield an average $\delta^{13}C$ value of –45.3‰ with a range from –22.2 to –65.3‰ (n = 121). Other hopanoids, including hopene and homohopanoic acid, are commonly widespread in modern-Holocene lake sediments, and they also exhibit a similar isotopic signature, ranging from –27.0 to –71.9‰ (n = 162).

The values of hopanoid $\delta^{13}C$ can also be very different within a single lake and between different lakes. For ancient lacustrine sequences, a relatively positive $\delta^{13}C$ dataset was obtained from the Jianghan Basin, China, with C_{30} 17α,21β-hopane $\delta^{13}C$ values ranging from –22.2 to –25.6‰ (average value of –24.0‰; n = 9)[25]. In contrast, the C_{29} and C_{30} 17α,21β-hopanes from the Eocene Green River Formation (USA) were found to be highly ^{13}C-depleted, ranging from –43.5 to –65.0‰ (average value of –51.8‰; n = 58)[24]. Much higher $\delta^{13}C$ values were also reported from this sequence (–26.7‰ and –32.8‰ for C_{29} and C_{30} 17α,21β-hopanes, respectively)[26]. In some modern-Holocene lake systems, hopanoids are characterized by relatively higher $\delta^{13}C$ values, for example, in Lake Sarbsko from Poland (C_{30} 17β,21β-hopane $\delta^{13}C$ ranging –36.3 to –38.6‰; n = 4)[27] and Ace Lake from Antarctica (diploptene $\delta^{13}C$ values ranging from –27 to –35.7‰; n = 5)[28], whereas the hopanoids from Lake Rotsee (Switzerland; diploptene and 17β,21β-homohopanoic acid)[21], Lake Koucha (China; diploptene and moretene)[29], Lake Bled (NW Slovenia; hop-17(21)-ene and diploptene)[30], and Lake Qalluuraq (Arctic Alaska; hop-17(21)-ene)[31] all show more negative

$\delta^{13}C$ values ranging from –42.3 to –71.9‰. Additionally, hopanoid $\delta^{13}C$ values are not always very negative (< –50‰) in a single lake, and relatively higher values (> –40‰) may coexist, such as those in the Alaskan thermokarst lake[32], Lake Tswaing from South Africa[33], and Lake Wudalianchi from China[22].

Crucially, in some lake settings (e.g., Lake Rotsee[21] and Green River Formation[24]), hopanoid $\delta^{13}C$ values are strongly ^{13}C-depleted (lower than –40‰) and up to 10‰ more negative relative to the co-occurring bulk organic matter or *n*-alkanes (ca. –30‰). In such situations, it is clear that active methanotrophy occurred in the water column and the hopanoids were largely derived from aerobic methanotrophic bacteria. In contrast, hopanoid $\delta^{13}C$ values higher than –40‰ suggest a dominant contribution from heterotrophic bacteria (e.g., Ace Lake from Antarctica[28] and lacustrine sequences from the Jianghan Basin[25]), which can also produce hopanoids and are related to relatively positive C isotopic signatures[34]. The data compilation suggests that –40‰ can be considered a baseline for evaluating the substantial contributions of methanotrophs (Supplementary Fig. 8). Based on a C isotopic mass-balance calculation (see Methods)[21], the proposed baseline for hopanoid $\delta^{13}C$ values of –40‰ indicates that approximately 10–20% of these compounds are derived from methanotrophic bacteria. In addition, hopanoid $\delta^{13}C$ values of –60‰ corresponds to approximately 40–70% of hopanoids originating from methanotrophs (Supplementary Fig. 8).

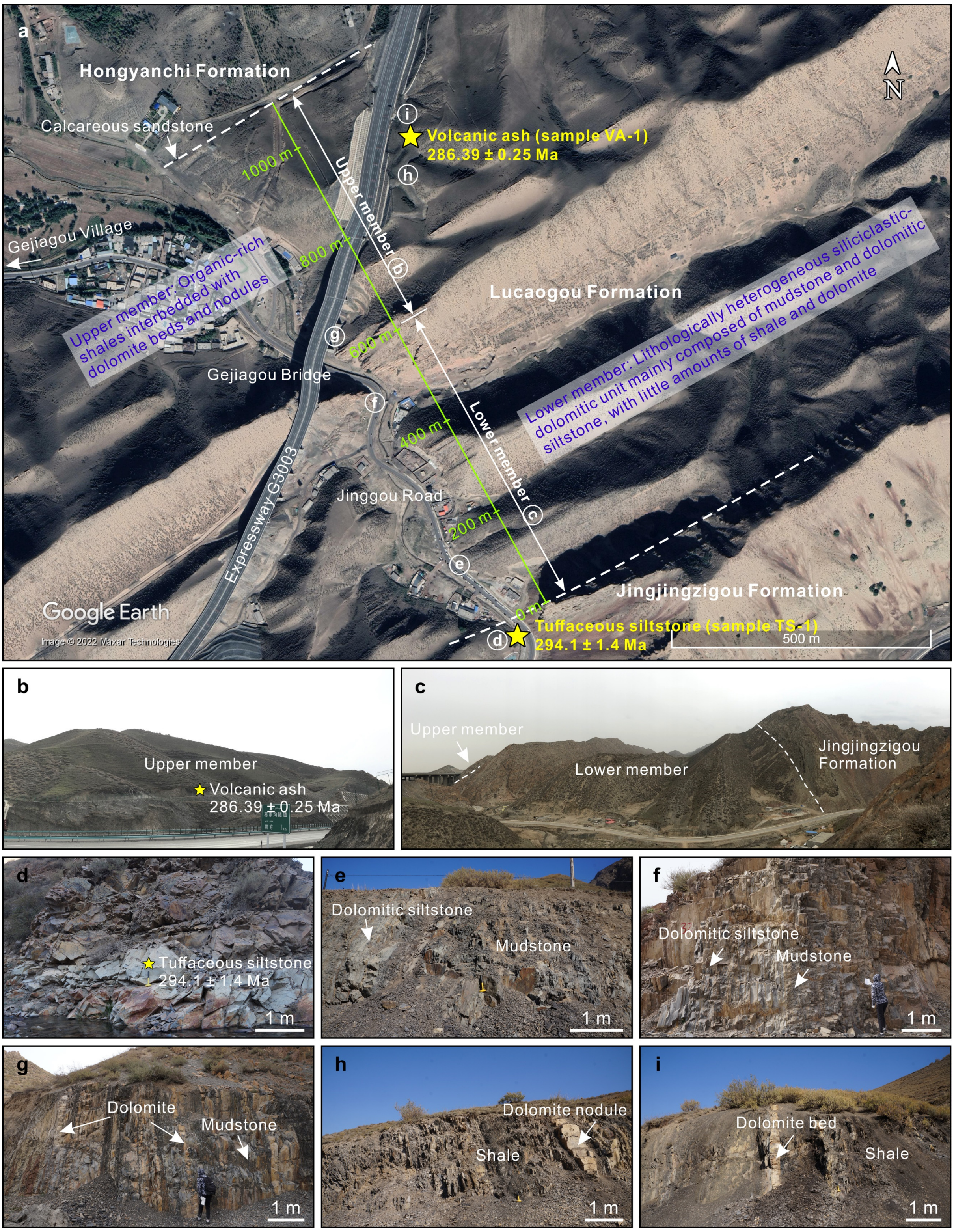

Supplementary Fig. 1 Location map and field photographs of the Jingjingzigou section. The outcrop is located in the Urumqi area, Xinjiang, NW China (GPS coordinates: 43°47′30″ N, 87°45′12″ E; altitude of approximately 1000 m; temperature continental climate), ~1.5 km southeast of Gejiagou Village. The Lucaogou Formation (Fm.) strata are nearly upright and

have a total thickness of ~1100 m. (**a**) Google Earth map showing the details of the Lucaogou Fm. with stratigraphic column heights (base map from Google Earth, Image © 2022 Maxar Technologies, https://earth.google.com/web/). Yellow stars represent the locations of the samples for zircon U-Pb dating. The volcanic ash sample (VS-1) was collected from the upper Lucaogou Fm., located at a height of ~925 m (also see Fig. 3). The tuffaceous siltstone (TS-1) was sampled from the uppermost part of the Jingjingzigou Fm., located ~30 m below the base of the Lucaogou Fm. White cycles mark the locations of field views as shown in (**b**–**i**). (**b**) Outcrop photograph showing the overview of the upper member of the Lucaogou Fm. The yellow star points to the accurate location of volcanic ash bed (see Fig. 2a for a close-up view). (**c**) Outcrop photograph showing the overview of the lower member of the Lucaogou Fm. (**d**) Gray and massive tuffaceous siltstone from the uppermost part of the underlying Jingjingzigou Fm. (**e**) and (**f**) Lithologically heterogeneous and mixed siliciclastic–dolomitic unit primarily comprising mudstone and dolomitic siltstone, with little amounts of shale and dolomite; they occur as few centimeters to decimeters-thick beds. (**g**) Grey-black mudstone–shale interval with dolomite interbeds. (**h**) and (**i**) Organic-rich shale intervals interbedded with decimeter-thick dolomite beds and nodules.

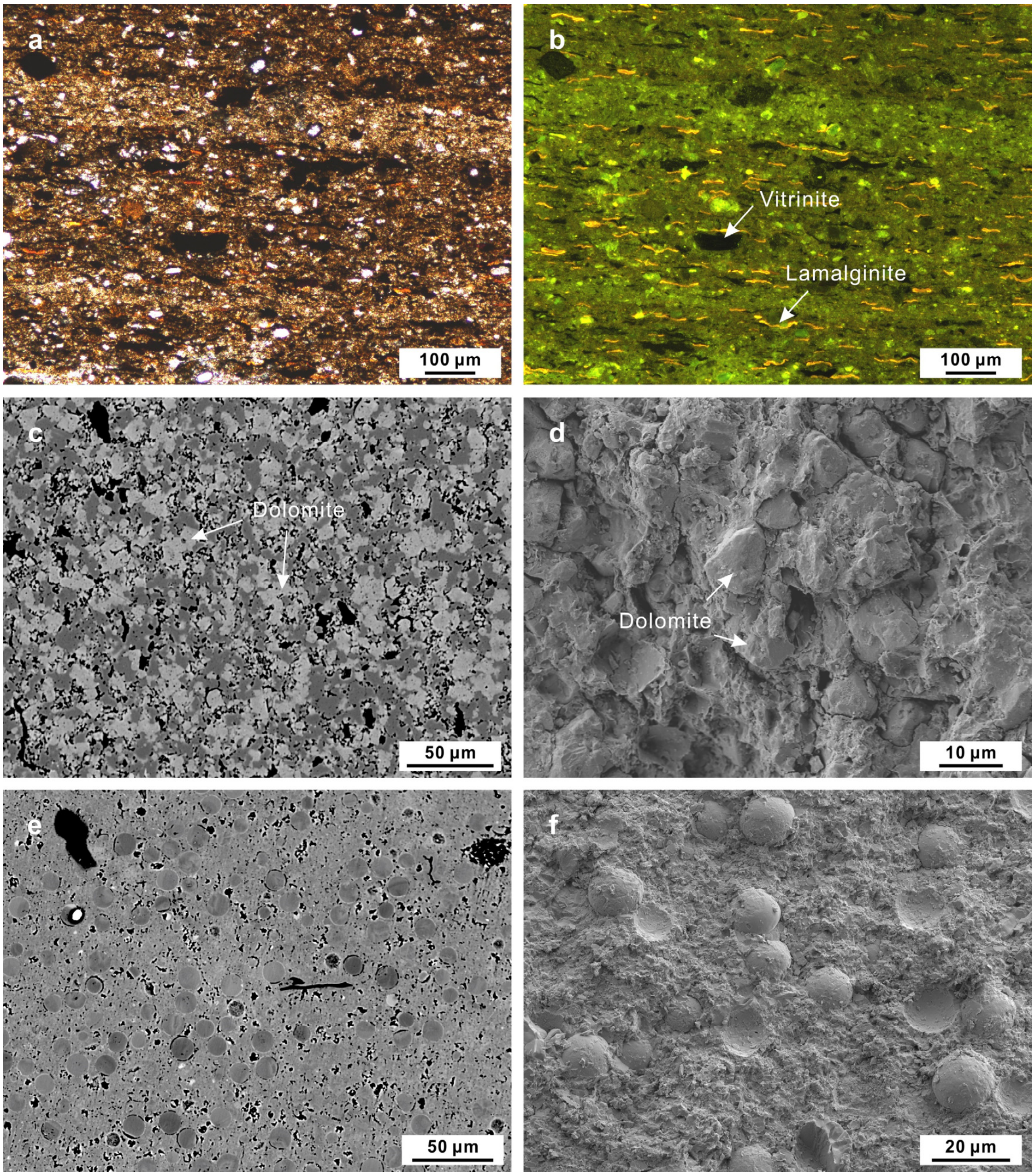

Supplementary Fig. 2 Photomicrographs of the typical lithologies in the Lucaogou Formation. (**a**) and (**b**) Representative organic-rich shale at a height of 1,038.08 m, showing lamination, lamalginites, and plant debris. (**c**) and (**d**) Dolomitic siltstone at a height of 313.33 m, showing fine-grained dolomite crystals embedded in a silicate mineral matrix. (**e**) and (**f**) Micritic dolomite at a height of 1,068.03 m, showing large amounts of microspheres composed of dolomite. Images produced via a transmitted light microscope (**a**), fluorescence-inducing blue light (**b**), and a scanning electron microscope in backscattered electron mode (**c**,**e**) and secondary electron mode (**d**,**f**).

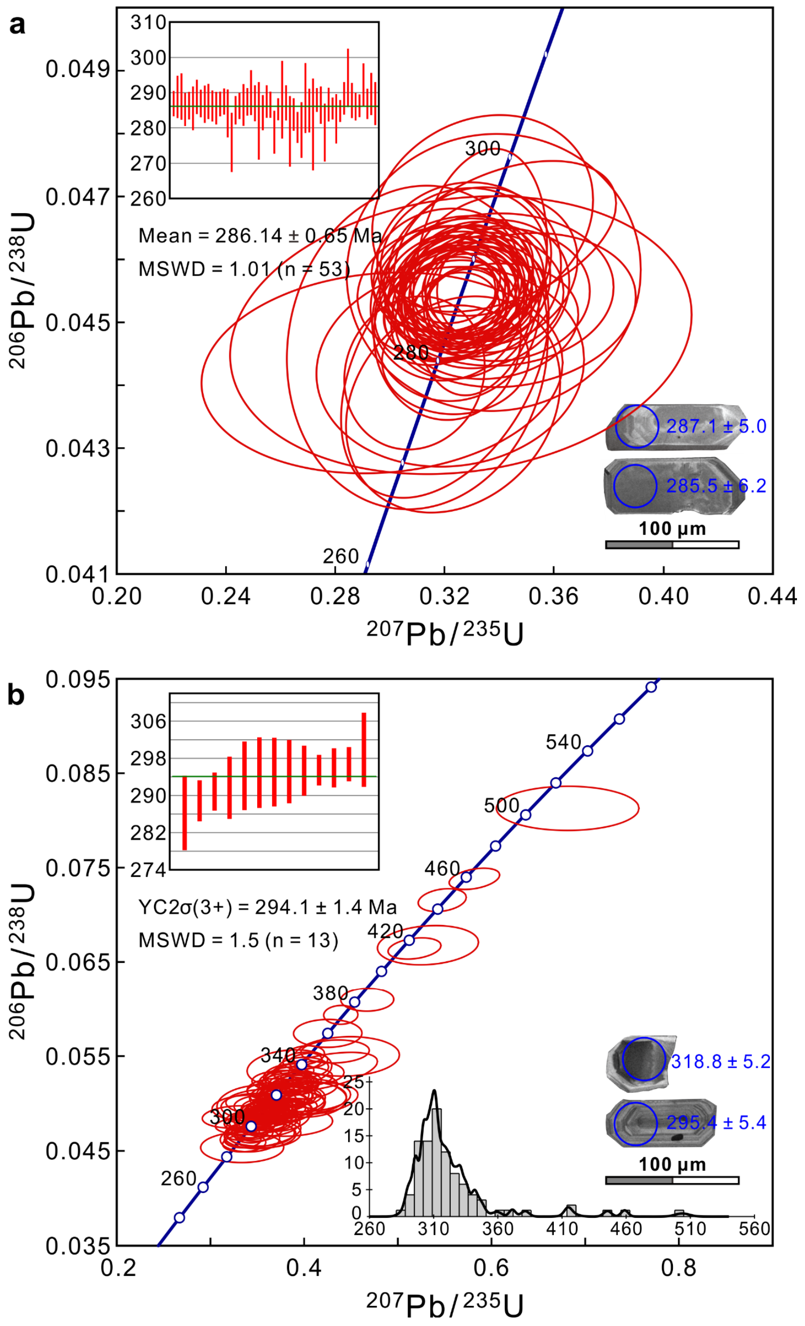

Supplementary Fig. 3 U-Pb Concordia diagrams, weighted mean age of a cluster ($^{206}Pb/^{238}U$ ages), and representative CL images of zircons from the Jingjingzigou outcrop, Xinjiang, NW China. (**a**) Sample VA-1 (volcanic ash; igneous zircons) from the upper member of the Lucaogou Formation, with a weighted mean $^{206}Pb/^{238}U$ age of 286.14 ± 0.65 Ma (2σ; MSWD = 1.01). (**b**) Sample TS-1 (tuffaceous siltstone; detrital zircons) from the uppermost Jingjingzigou Formation, with a YC2σ(3+) age of 294.1 ± 1.4 Ma (2σ; MSWD = 1.5). YC2σ(3+) = mean age of the youngest three or more grains that overlap in age at 2σ.

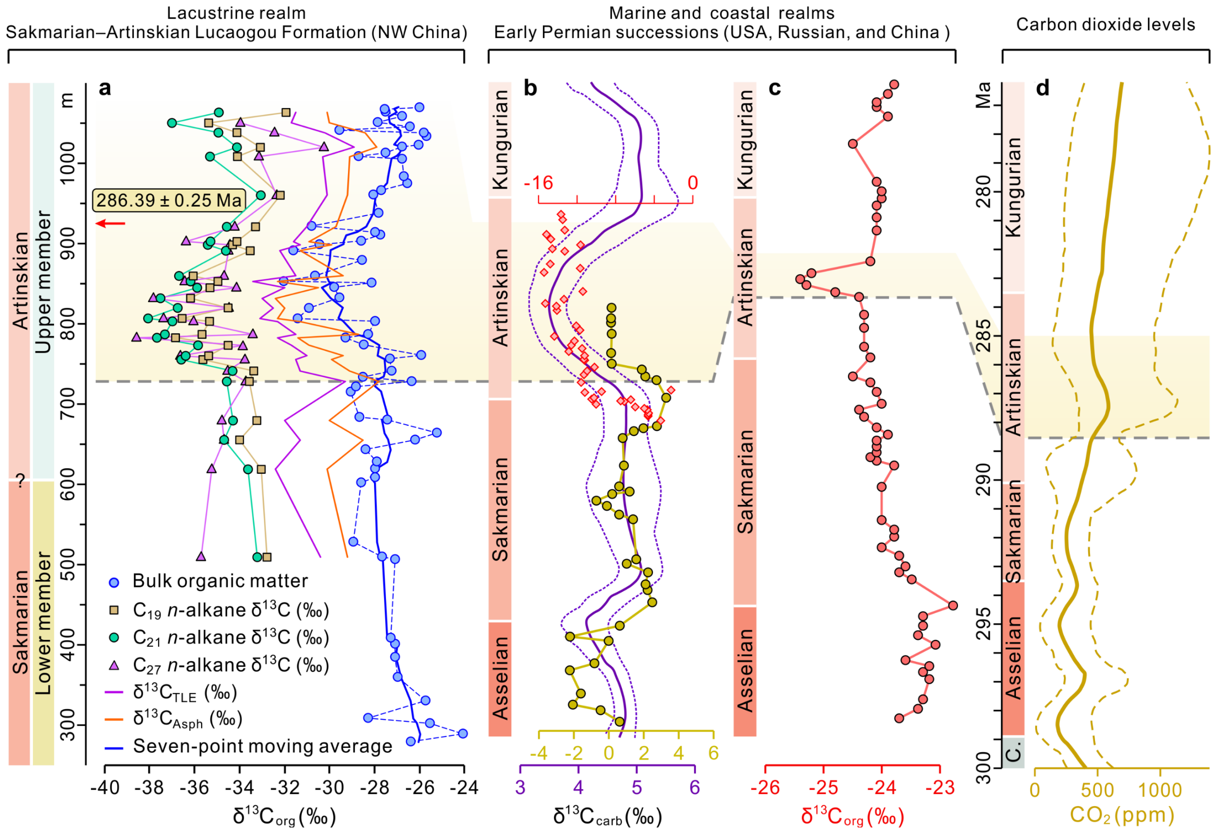

Supplementary Fig. 4 Carbon isotope stratigraphic comparison and global atmospheric CO_2 levels. (**a**) $\delta^{13}C_{org}$ profiles of the bulk organic matter, *n*-alkanes, total lipid extract (TLE), and asphaltene (Asph) show a negative excursion within the upper part of the lacustrine Lucaogou Formation (Junggar Basin, NW China). The zircon U-Pb CA-ID-TIMS age from the upper member is of 286.39 ± 0.25 Ma. (**b**) $\delta^{13}C_{carbonate}$ data for marine brachiopod shells from the U.S. Craton and the Russian Platform[17] (solid curve) and those for carbonates from the Zhongdi succession, South China[6] (orange circle) and the Dal'ny Tulkas section in the southern Urals, Russia[20] (red diamond). (**c**) Bulk $\delta^{13}C_{org}$ data for a coastal succession from the Yuzhou section, North China[18]. The negative CIEs during the mid-Artinskian are highlighted by the yellow shaded field in (**a**–**c**). (**d**) Reconstructed global atmospheric partial pressure of CO_2 (pCO_2) curve (75% confidence interval) during the Early Permian[19].

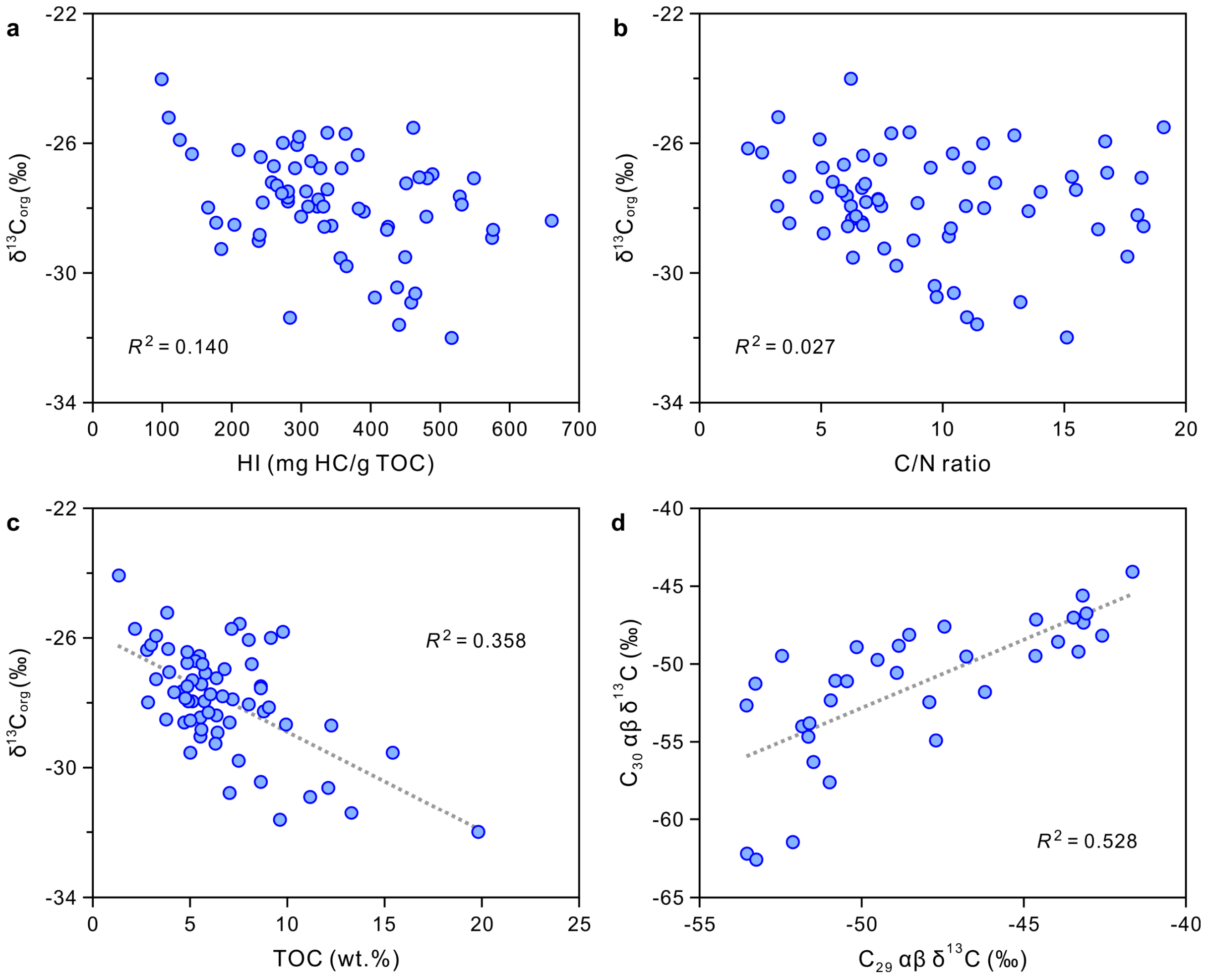

Supplementary Fig. 5 Bulk organic C isotopes, TOC values, C/N ratios, and hopane C isotopes for the Lucaogou shales. (**a**) Cross-plot of the bulk $\delta^{13}C_{org}$ vs. hydrogen index (HI). (**b**) Cross-plot of the bulk $\delta^{13}C_{org}$ vs. C/N ratio. (**c**) Cross-plot of the bulk $\delta^{13}C_{org}$ vs. total organic carbon (TOC). (**d**) Cross-plot of C_{30} αβ hopane δ^{13}C vs. C_{29} αβ hopane δ^{13}C.

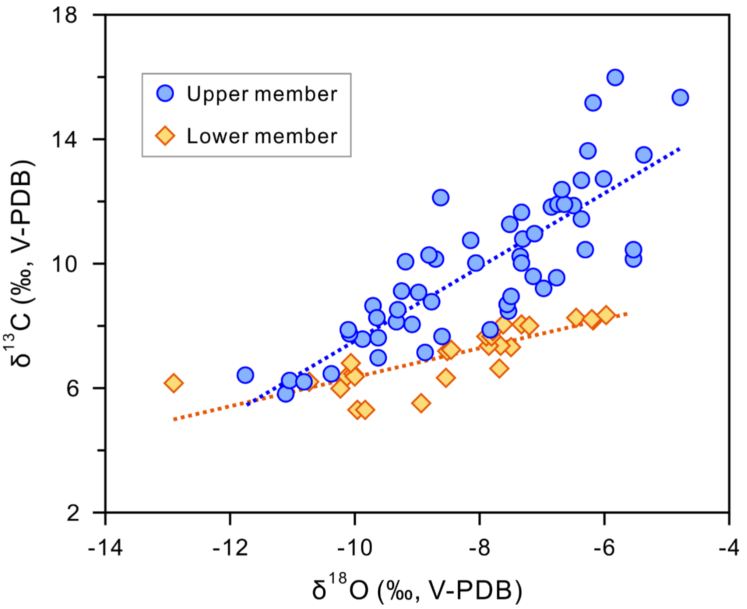

Supplementary Fig. 6 Stable C and O isotope data for the Lucaogou dolomite.

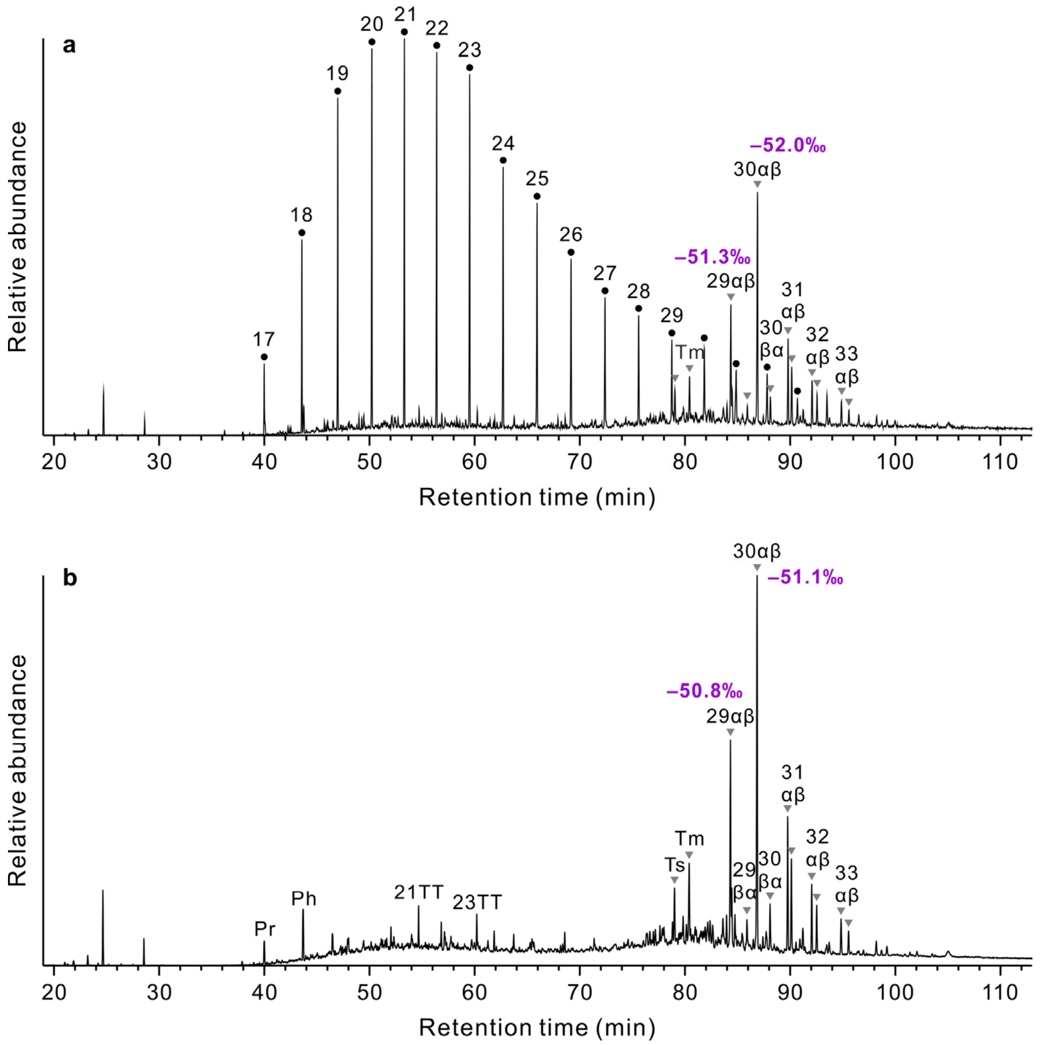

Supplementary Fig. 7 Representative total ion chromatograms of the saturated hydrocarbon fraction isolated from the Lucaogou shale. (**a**) Before ZSM-5 molecular sieve treatment. (**b**) After ZSM-5 treatment. The sample was collected at a height of 898.61 m, showing negative hopane $\delta^{13}C$ values. No isotopic fractionation occurred during molecular sieving. Black circles represent the *n*-alkanes and gray triangles represent the hopanes, with the stereochemistry shown above. Numbers indicate the number of C atoms. Pr = pristane; Ph = phytane; 21 and 23TT = C_{21} and C_{23} tricyclic terpanes, respectively; Ts = C_{27} 18α(H)-22,29,30-trisnorneohopane; Tm = C_{27} 17α(H)-22,29,30-trisnorhopane; 29αβ = C_{29} 17α(H),21β(H)-norhopane; 29βα = C_{29} 17β(H),21α(H)-norhopane; 30αβ = C_{30} 17α(H),21β(H)-hopane; 30βα = C_{30} 17β(H),21α(H)-hopane; and 31–33αβ = C_{31}–C_{33} 17α(H),21β(H),22S (and R) homohopanes.

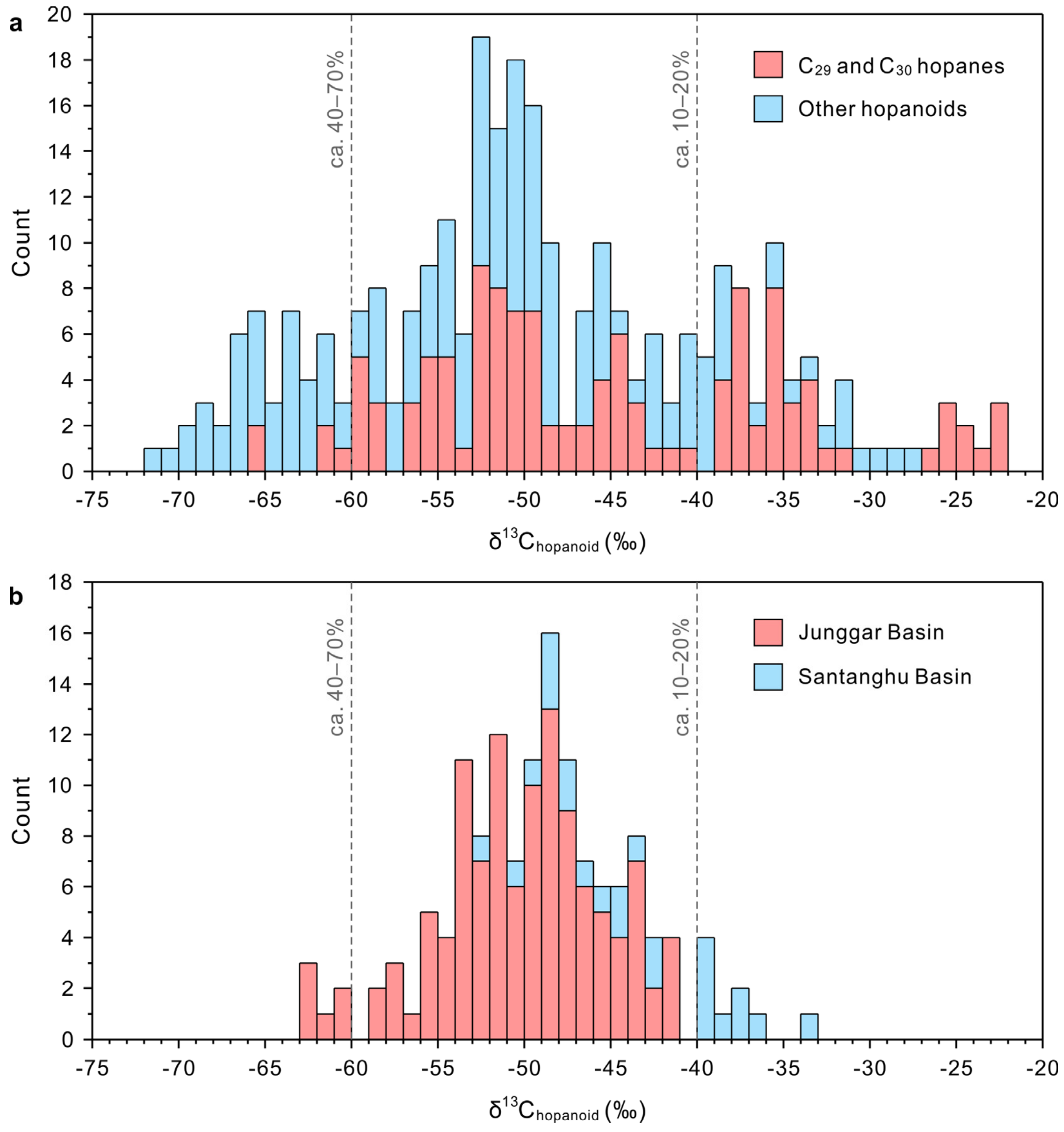

Supplementary Fig. 8 Compilation of hopanoid $\delta^{13}C$ values in lake systems. (**a**) C_{29} and C_{30} hopanes and other hopanoids (hop-17(21)-ene, diploptene, neohop-13(18)-ene, moretene, and homohopanoic acid) from modern-Holocene[21,22,27–33,35,36], Miocene[37,38], Oligocene[39], and Eocene[23–26,40–43] lakes. (**b**) C_{29} and C_{30} hopanes from paleo-Lake Junggar in this study. Data from the Santanghu Basin were obtained from ref. [44]. The gray-dashed lines and numbers denote the contribution of aerobic methanotrophs to hopanoids calculated using a C isotopic mass-balance equation[21].

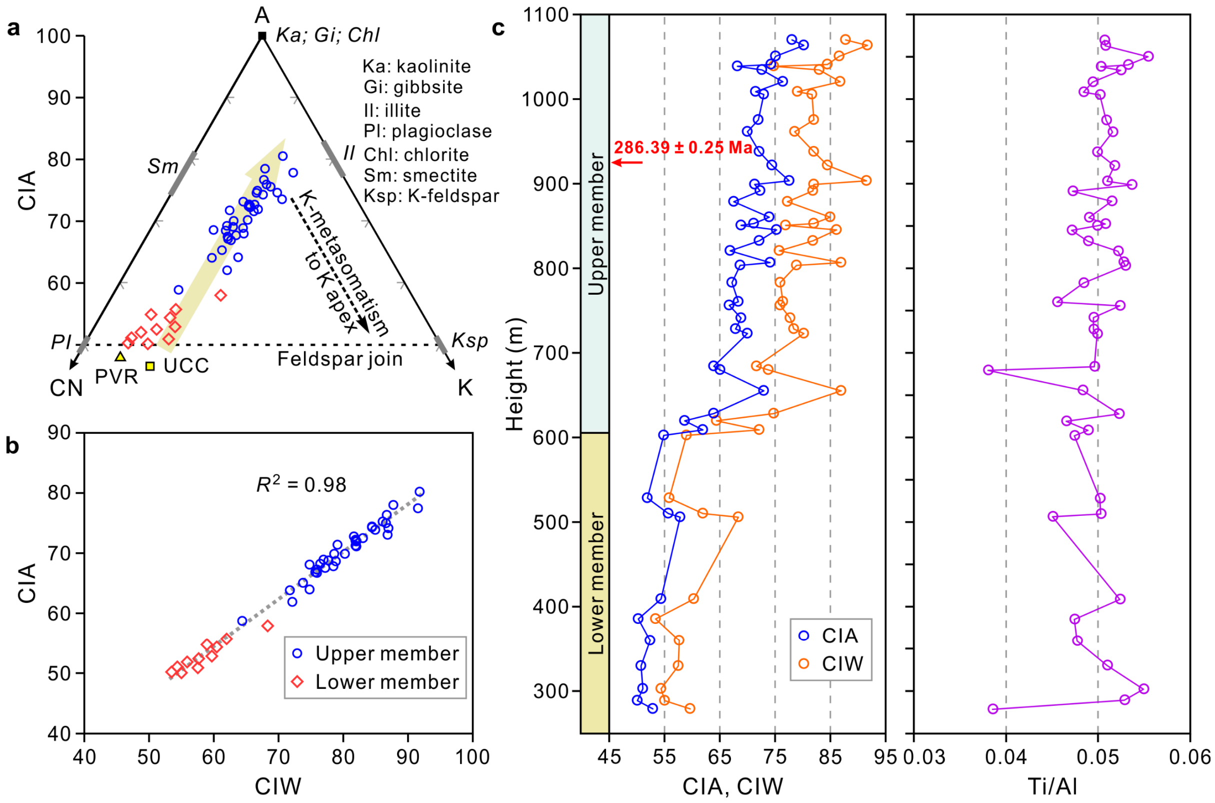

Supplementary Fig. 9 Chemical weathering indices for the Lucaogou shales. (**a**) A-CN-K diagram with the chemical index of alteration (CIA) scale on the left (after ref. [7]). The yellow square and triangle denote the compositions of the average upper continental crust (UCC)[45] and Late Paleozoic volcanic rocks in the Junggar Basin (PVR)[8], respectively. The green solid arrow and black-dashed arrow represent the predicted weathering trend and the trend affected by K-metasomatism, respectively. (**b**) Cross-plot of CIA vs. chemical index of weathering (CIW). (**c**) Stratigraphic variations in the CIA, CIW, and Ti/Al ratio values.

Supplementary References

1. Wu, H. et al. A unique lacustrine mixed dolomitic-clastic sequence for tight oil reservoir within the middle Permian Lucaogou Formation of the Junggar Basin, NW China: Reservoir characteristics and origin. *Mar. Pet. Geol.* **76**, 115–132 (2016).
2. Sun, F. et al. Methanogen microfossils and methanogenesis in Permian lake deposits. *Geology* **49**, 13–18 (2021).
3. Carroll, A. R. Upper Permian lacustrine organic facies evolution, Southern Junggar Basin, NW China. *Org. Geochem.* **28**, 649–667 (1998).
4. Liang, J. et al. Formation conditions of Jimusaer oil shale at the northern foot of Bogda Mountain, China. *Oil Shale* **31**, 19–29 (2014).
5. Zhu, H.-C., Ouyang, S., Zhan, J.-Z. & Wang, Z. Comparison of Permian palynological assemblages from the Junggar and Tarim Basins and their phytoprovincial significance. *Rev. Palaeobot. Palynol.* **136**, 181–207 (2005).
6. Buggisch, W., Wang, X., Alekseev, A. S. & Joachimski, M. M. Carboniferous–Permian carbon isotope stratigraphy of successions from China (Yangtze platform), USA (Kansas) and Russia (Moscow Basin and Urals). *Paleogeogr. Paleoclimatol. Paleoecol.* **301**, 18–38 (2011).
7. Fedo, C. M., Nesbitt, H. W. & Young, G. M. Unraveling the effects of potassium metasomatism in sedimentary rocks and paleosols, with implications for paleoweathering conditions and provenance. *Geology* **23**, 921–924 (1995).
8. Zheng, J., Sun, M., Zhao, G., Robinson, P. T. & Wang, F. Elemental and Sr–Nd–Pb isotopic geochemistry of Late Paleozoic volcanic rocks beneath the Junggar basin, NW China: Implications for the formation and evolution of the basin basement. *J. Asian Earth Sci.* **29**, 778–794 (2007).
9. Yang, J., Cawood, P. A., Du, Y., Feng, B. & Yan, J. Global continental weathering

trends across the Early Permian glacial to postglacial transition: Correlating high- and low-paleolatitude sedimentary records. *Geology* **42**, 835–838 (2014).

10. Scheffler, K., Hoernes, S. & Schwark, L. Global changes during Carboniferous–Permian glaciation of Gondwana: Linking polar and equatorial climate evolution by geochemical proxies. *Geology* **31**, 605–608 (2003).
11. Korte, C., Jasper, T., Kozur, H. W. & Veizer, J. $\delta^{18}O$ and $\delta^{13}C$ of Permian brachiopods: A record of seawater evolution and continental glaciation. *Paleogeogr. Paleoclimatol. Paleoecol.* **224**, 333–351 (2005).
12. Korte, C., Jones, P. J., Brand, U., Mertmann, D. & Veizer, J. Oxygen isotope values from high-latitudes: Clues for Permian sea-surface temperature gradients and Late Palaeozoic deglaciation. *Paleogeogr. Paleoclimatol. Paleoecol.* **269**, 1–16 (2008).
13. Marchetti, L. et al. The Artinskian Warming Event: an Euramerican change in climate and the terrestrial biota during the early Permian. *Earth-Sci. Rev.* **226**, 103922 (2022).
14. Lewan, M. D. Effects of thermal maturation on stable organic carbon isotopes as determined by hydrous pyrolysis of Woodford Shale. *Geochim. Cosmochim. Acta* **47**, 1471–1479 (1983).
15. Lu, J. et al. Volcanically driven lacustrine ecosystem changes during the Carnian Pluvial Episode (Late Triassic). *Proc. Natl Acad. Sci. USA* **118**, e2109895118 (2021).
16. Meyers, P. A. Organic geochemical proxies of paleoceanographic, paleolimnologic, and paleoclimatic processes. *Org. Geochem.* **27**, 213–250 (1997).
17. Grossman, E. L. et al. Glaciation, aridification, and carbon sequestration in the Permo-Carboniferous: The isotopic record from low latitudes. *Paleogeogr. Paleoclimatol. Paleoecol.* **268**, 222–233 (2008).
18. Lu, J. et al. Records of organic carbon isotopic composition ($\delta^{13}C_{org}$) and volcanism linked to changes in atmospheric pCO_2 and climate during the Late Paleozoic Icehouse.

Glob. Planet. Change **207**, 103654 (2021).

19. Richey, J. D. et al. Influence of temporally varying weatherability on CO_2-climate coupling and ecosystem change in the late Paleozoic. *Clim. Past* **16**, 1759–1775 (2020).
20. Zeng, J., Cao, C.-Q., Davydov, V. I. & Shen, S.-Z. Carbon isotope chemostratigraphy and implications of palaeoclimatic changes during the Cisuralian (Early Permian) in the southern Urals, Russia. *Gondwana Res.* **21**, 601–610 (2012).
21. Naeher, S. et al. Tracing the methane cycle with lipid biomarkers in Lake Rotsee (Switzerland). *Org. Geochem.* **66**, 174–181 (2014).
22. Yao, Y. et al. Permafrost thaw induced abrupt changes in hydrology and carbon cycling in Lake Wudalianchi, northeastern China. *Geology* **49**, 1117–1121 (2021).
23. Collister, J. W., Summons, R. E., Lichtfouse, E. & Hayes, J. M. An isotopic biogeochemical study of the Green River oil shale. *Org. Geochem.* **19**, 265–276 (1992).
24. Collister, J. W. & Wavrek, D. A. $\delta^{13}C$ compositions of saturate and aromatic fractions of lacustrine oils and bitumens: evidence for water column stratification. *Org. Geochem.* **24**, 913–920 (1996).
25. Grice, K., Schouten, S., Peters, K. E. & Damsté, J. S. S. Molecular isotopic characterisation of hydrocarbon biomarkers in Palaeocene–Eocene evaporitic, lacustrine source rocks from the Jianghan Basin, China. *Org. Geochem.* **29**, 1745–1764 (1998).
26. Ruble, T. E., Bakel, A. J. & Philp, R. P. Compound specific isotopic variability in Uinta Basin native bitumens: paleoenvironmental implications. *Org. Geochem.* **21**, 661–671 (1994).
27. Woszczyk, M. et al. Composition and origin of organic matter in surface sediments of Lake Sarbsko: A highly eutrophic and shallow coastal lake (northern Poland). *Org. Geochem.* **42**, 1025–1038 (2011).
28. Schouten, S. et al. Molecular organic tracers of biogeochemical processes in a saline

meromictic lake (Ace Lake). *Geochim. Cosmochim. Acta* **65**, 1629–1640 (2001).

29. Aichner, B., Wilkes, H., Herzschuh, U., Mischke, S. & Zhang, C. Biomarker and compound-specific $\delta^{13}C$ evidence for changing environmental conditions and carbon limitation at Lake Koucha, eastern Tibetan Plateau. *J. Paleolimn.* **43**, 873–899 (2010).
30. Petrišič, M. G., Heath, E. & Ogrinc, N. Lipid biomarkers and their stable carbon isotopes in oxic and anoxic sediments of Lake Bled (NW Slovenia). *Geomicrobiol. J.* **34**, 606–617 (2017).
31. Elvert, M. et al. Methane turnover and environmental change from Holocene lipid biomarker records in a thermokarst lake in Arctic Alaska. *Holocene* **26**, 1766–1777 (2016).
32. Davies, K. L. et al. Diploptene $\delta^{13}C$ values from contemporary thermokarst lake sediments show complex spatial variation. *Biogeosciences* **13**, 2611–2621 (2016).
33. Kristen, I. et al. Biomarker and stable carbon isotope analyses of sedimentary organic matter from Lake Tswaing: evidence for deglacial wetness and early Holocene drought from South Africa. *J. Paleolimn.* **44**, 143–160 (2010).
34. Pancost, R. D. & Damsté, J. S. S. Carbon isotopic compositions of prokaryotic lipids as tracers of carbon cycling in diverse settings. *Chem. Geol.* **195**, 29–58 (2003).
35. Spooner, N. et al. Stable carbon isotopic correlation of individual biolipids in aquatic organisms and a lake bottom sediment. *Org. Geochem.* **21**, 823–827 (1994).
36. Uemura, H. & Ishiwatari, R. Identification of unusual 17β(H)-moret-22(29)-ene in lake sediments. *Org. Geochem.* **23**, 675–680 (1995).
37. Huang, Y., Lockheart, M. J., Collister, J. W. & Eglinton, G. Molecular and isotopic biogeochemistry of the Miocene Clarkia Formation: hydrocarbons and alcohols. *Org. Geochem.* **23**, 785–801 (1995).
38. Huang, Y., Lockheart, M. J., Logan, G. A. & Eglinton, G. Isotope and molecular

evidence for the diverse origins of carboxylic acids in leaf fossils and sediments from the Miocene Lake Clarkia deposit, Idaho, USA. *Org. Geochem.* **24**, 289–299 (1996).

39. Bechtel, A., Hámor-Vidó, M., Gratzer, R., Sachsenhofer, R. F. & Püttmann, W. Facies evolution and stratigraphic correlation in the early Oligocene Tard Clay of Hungary as revealed by maceral, biomarker and stable isotope composition. *Mar. Petrol. Geol.* **35**, 55–74 (2012).
40. Freeman, K. H., Hayes, J. M., Trendel, J.-M. & Albrecht, P. Evidence from carbon isotope measurements for diverse origins of sedimentary hydrocarbons. *Nature* **343**, 254–256 (1990).
41. Duan, Y., Wu, B., Zheng, G., Zhang, H. & Zheng, C. The specific carbon isotopic compositions of branched and cyclic hydrocarbons from Fushun oil shale. *Chin. Sci. Bull.* **49**, 369–373 (2004).
42. Volkman, J. K., Zhang, Z., Xie, X., Qin, J. & Borjigin, T. Biomarker evidence for *Botryococcus* and a methane cycle in the Eocene Huadian oil shale, NE China. *Org. Geochem.* **78**, 121–134 (2015).
43. Niu, C. et al. Origin and geochemical implications of hopanoids in saline lacustrine crude oils from Huanghekou East Sag and Laizhouwan Northeastern Sag, Bohai Bay Basin. *ACS Omega* **6**, 30298–30314 (2021).
44. Liu, B. et al. Depositional environment of oil shale within the second member of Permian Lucaogou Formation in the Santanghu Basin, Northwest China. *Int. J. Coal Geol.* **175**, 10–25 (2017).
45. Taylor, S. R. & McLennan, S. M. *The Continental Crust: Its Composition and Evolution* (Blackwell Scientific, Oxford, 1985).
