# Supplementary material for: Sustained and intensified lacustrine methane cycling during Early Permian climate warming
Source: Nat Commun. 2022 Aug 18;13:4856. doi: 10.1038/s41467-022-32438-2 (PMC9388690; doi:10.1038/s41467-022-32438-2)
Supplement: Supplementary file 2 — Description of Additional Supplementary Files [file 41467_2022_32438_MOESM2_ESM.pdf]

Description of Additional Supplementary Files

File Name: Supplementary Data 1
Description: LA-ICP-MS zircon U-Pb date for volcanic ash (sample VA-1) and tuffaceous siltstone (sample TS-1) from the Jingjingzigou outcrop

File Name: Supplementary Data 2
Description: U-Pb isotopic data for CA-ID-TIMS zircon analyses from sample VA-1, upper Lucaogou Formation

File Name: Supplementary Data 3
Description: Bulk organic C isotopes, TOC, and TN values, and Rock-Eval pyrolysis data for the Lucaogou shales

File Name: Supplementary Data 4
Description: Compound-specific C isotope data and biomarker maturity parameters for the Lucaogou shales

File Name: Supplementary Data 5
Description: Stable C and O isotope data for the Lucaogou dolomite

File Name: Supplementary Data 6
Description: Compound-specific C isotope dataset of hopanoids from Cenozoic lake systems

File Name: Supplementary Data 7
Description: Whole-rock major elemental compositions, chemical weathering indices, and calculated land surface temperature data

File Name: Supplementary Data 8
Description: Overview of the Sakmarian–Artinskian (Early Permian) lake systems
