# Supplementary material for: Sustained and intensified lacustrine methane cycling during Early Permian climate warming
Source: Nat Commun. 2022 Aug 18;13:4856. doi: 10.1038/s41467-022-32438-2 (PMC9388690; doi:10.1038/s41467-022-32438-2)
Supplement: Supplementary file 11 — Reporting Summary [file 41467_2022_32438_MOESM11_ESM.pdf]

nature portfolio

Corresponding author(s): Wenxuan Hu

Last updated by author(s): Jul 17, 2022

Reporting Summary

Nature Portfolio wishes to improve the reproducibility of the work that we publish. This form provides structure for consistency and transparency in reporting. For further information on Nature Portfolio policies, see our Editorial Policies and the Editorial Policy Checklist.

Statistics

For all statistical analyses, confirm that the following items are present in the figure legend, table legend, main text, or Methods section.

n/a	Confirmed	
☐	☒	The exact sample size (*n*) for each experimental group/condition, given as a discrete number and unit of measurement
☐	☒	A statement on whether measurements were taken from distinct samples or whether the same sample was measured repeatedly
☒	☐	The statistical test(s) used AND whether they are one- or two-sided<br>*Only common tests should be described solely by name; describe more complex techniques in the Methods section.*
☐	☒	A description of all covariates tested
☒	☐	A description of any assumptions or corrections, such as tests of normality and adjustment for multiple comparisons
☐	☒	A full description of the statistical parameters including central tendency (e.g. means) or other basic estimates (e.g. regression coefficient) AND variation (e.g. standard deviation) or associated estimates of uncertainty (e.g. confidence intervals)
☒	☐	For null hypothesis testing, the test statistic (e.g. *F*, *t*, *r*) with confidence intervals, effect sizes, degrees of freedom and *P* value noted<br>*Give P values as exact values whenever suitable.*
☒	☐	For Bayesian analysis, information on the choice of priors and Markov chain Monte Carlo settings
☒	☐	For hierarchical and complex designs, identification of the appropriate level for tests and full reporting of outcomes
☒	☐	Estimates of effect sizes (e.g. Cohen's *d*, Pearson's *r*), indicating how they were calculated

Our web collection on statistics for biologists contains articles on many of the points above.

Software and code

Policy information about availability of computer code

Data collection	No software was used.
Data analysis	Iolite software package; ISOPLOT/Ex program (ver. 4.15); Tripoli and ET_Redux software; Microsoft Excel 2019

For manuscripts utilizing custom algorithms or software that are central to the research but not yet described in published literature, software must be made available to editors and reviewers. We strongly encourage code deposition in a community repository (e.g. GitHub). See the Nature Portfolio guidelines for submitting code & software for further information.

Data

Policy information about availability of data

All manuscripts must include a data availability statement. This statement should provide the following information, where applicable:

- Accession codes, unique identifiers, or web links for publicly available datasets
- A description of any restrictions on data availability
- For clinical datasets or third party data, please ensure that the statement adheres to our policy

All data supporting the findings of this study are included in the Supplementary Data files (Supplementary Data 1–8).

Field-specific reporting

Please select the one below that is the best fit for your research. If you are not sure, read the appropriate sections before making your selection.

☐ Life sciences ☐ Behavioural & social sciences ☒ Ecological, evolutionary & environmental sciences

For a reference copy of the document with all sections, see nature.com/documents/nr-reporting-summary-flat.pdf

Ecological, evolutionary & environmental sciences study design

All studies must disclose on these points even when the disclosure is negative.

Study description	A study reports the occurrence of sustained and intensified microbial CH4 cycling in paleo-Lake Junggar in northwestern China, one of the largest known Phanerozoic lakes, during Early Permian climate warming.
Research sample	The samples analyzed in this study were collected from the Jingjingzigou section (43°47′30″ N, 87°45′12″ E) in the Urumqi area, Xinjiang, NW China.
Sampling strategy	Sample were collected for petrographic, zircon U-Pb and geochemical analyses. The samples were visually examined to ensure that they were fresh. The sample size was deemed sufficient for above analyses.
Data collection	Microscopic analysis, organic element and Rock-Eval pyrolysis analyses, bulk organic C isotope, carbonate C-O isotope analysis, XRF analysis were conducted at Nanjing University; LA-ICP-MS analysis was conducted at Nanjing Hongchuang Exploration Technology Service Co., Ltd.; CA-ID-TIMS analysis was conducted at the Massachusetts Institute of Technology Isotope Laboratory; and GC-MS and GC-IRMS analyses were conducted at the State Key Laboratory of Shale Oil and Gas Enrichment Mechanisms and Effective Development of SINOPEC.
Timing and spatial scale	All samples were collected between 2018 and 2020 from the Jingjingzigou section. The Lucaogou Formation strata are nearly upright and have a total thickness of ~1100 m.
Data exclusions	No data were excluded from analyses.
Reproducibility	The reproducibility and accuracy were evaluated by measuring standards between sample measurements or measured in duplicate.
Randomization	This was not involved in this study.
Blinding	This was not involved in this study.

Did the study involve field work? ☒ Yes ☐ No

Field work, collection and transport

Field conditions	The field site is located in the temperate continental climate zone. The altitude is of approximately 1000 m. Climate is arid and hot in the field season (summertime) . The outcrop mainly occurs along road cuts, which are generally continuous.
Location	The Jingjingzigou section is located at the Urumqi area, Xinjiang, NW China (GPS coordinates: 43°47′30″ N, 87°45′12″ E), ~1.5 km southeast of Gejiagou Village.
Access & import/export	Samples were collected from a non-protected site in a responsible manner and in compliance with the local and national laws.
Disturbance	No disturbance was caused during collection.

Reporting for specific materials, systems and methods

We require information from authors about some types of materials, experimental systems and methods used in many studies. Here, indicate whether each material, system or method listed is relevant to your study. If you are not sure if a list item applies to your research, read the appropriate section before selecting a response.

Materials & experimental systems

n/a	Involved in the study
☒	☐ Antibodies
☒	☐ Eukaryotic cell lines
☒	☐ Palaeontology and archaeology
☒	☐ Animals and other organisms
☒	☐ Human research participants
☒	☐ Clinical data
☒	☐ Dual use research of concern

Methods

n/a	Involved in the study
☒	☐ ChIP-seq
☒	☐ Flow cytometry
☒	☐ MRI-based neuroimaging
